# Insights into eruption dynamics from TROPOMI/PlumeTraj-derived SO_2_ emissions during the 2022 eruption of Mauna Loa, Hawaiʻi

**DOI:** 10.1007/s00445-025-01839-8

**Published:** 2025-08-14

**Authors:** Ben Esse, Mike Burton, Hugues Brenot, Nicolas Theys

**Affiliations:** 1 COMET, Department of Earth and Environmental Sciences, The University of Manchester, Manchester, UK; 2 Royal Belgian Institute for Space Aeronomy, Brussels, Belgium

**Keywords:** Sulphur dioxide, Mauna Loa, TROPOMI, Fissure eruption

## Abstract

Volcanic fissure eruptions can produce voluminous gas emissions, posing a risk to local and distal populations and potentially impacting global climate. Quantifying the emission rate and altitude of injection of these emissions allows forecasting of impacts and provides key insights into the magma dynamics driving eruptions. Daily global observations from satellite instruments such as TROPOMI combined with trajectory modelling with PlumeTraj deliver these emission rate and altitude data. Here, we report satellite-derived SO_2_ emissions from the 2022 eruption of Mauna Loa, which lasted only 13 days but produced an SO_2_ plume that circled the globe, displaying a highly variable emission rate and injection altitude. Three key discoveries were made: we detect precursory SO_2_ emissions up to 3 h before the eruption start; peaks in emission rate are correlated with onset and cessation of activity at different fissures; the SO_2_ injection altitude was modulated by the available moisture content of the ambient air. We suggest that alignment of the fissure geometry with the wind direction could potentially explain how the initial emissions reached 14 km asl, approaching the tropopause. The total SO_2_ measured from this eruption is 600 (± 300) kt. These results demonstrate how satellite measurements can provide new insights into eruptive and degassing mechanisms and highlight that better constraints on the SO_2_ emissions from fissure eruptions globally are needed to understand their impact on climate.

**Supplementary Information:**

The online version contains supplementary material available at 10.1007/s00445-025-01839-8.

## Introduction

Mauna Loa, the largest active volcano in Hawaiʻi, erupted between 28 November and 10 December 2022 (Global Volcanism Program 2023; Lynn et al. 2024). It produced lava flows with a total volume of ~ 145 million m^3^ to the south-west and north-east of the summit crater (Dietterich et al., 2025), fortunately stopping ~ 3 km short of the Daniel K. Inouye State Highway, avoiding significant disruption to local communities. Mauna Loa poses a significant hazard to the population of Hawaiʻi, especially in the Kona region (on the western side of the island) which has been inundated by lava flows from 7 of the 40 eruptions since 1832, and most recently in 1950 (Gregg et al. 2004). As well as lava flows, volcanic gas emissions (including the phenomenon of volcanic smog or “vog”) are also a major hazard from volcanism in Hawaiʻi (e.g. Michaud et al. 2004; Longo et al. 2008, 2010; Whitty et al. 2020) and beyond (Schmidt et al. 2015; Ilyinskaya et al. 2017; Edmonds et al. 2018; Barsotti 2020). This highlights the need for robust monitoring for future eruptions from Mauna Loa, as well as a clear understanding of its eruptive mechanisms. Measurements of volcanic gas emission are a key element in volcano monitoring strategies, providing insights into the magmatic system (Allard et al. 2005; Burton et al. 2007; McCormick Kilbride et al. 2016; Barrière et al. 2019), strengthening eruption forecasting (Aiuppa et al. 2007, 2010) and informing plume dispersal forecasts (Boichu et al. 2014; Businger et al. 2015; Pfeffer et al. 2024). Gas plume dispersal from a complex fissure eruption such as Mauna Loa 2022 is controlled by multiple factors including gas injection altitude, which is a function of local meteorology and eruption style and vigour (Stothers et al. 1986; Woods 1993a, b; Kaminski et al. 2011).

In this paper, we present satellite-derived SO_2_ emissions from the 2022 eruption of Mauna Loa, analysing SO_2_ imagery from the TROPOspheric Monitoring Instrument (TROPOMI) with the PlumeTraj trajectory-analysis toolkit (Pardini et al. 2017; Queißer et al. 2019; Burton et al. 2021; Esse et al. 2023; Hayer et al. 2023). The initial plume reached altitudes approaching the tropopause (14 km above sea level) in the absence of any significant explosive activity. This highlights the potential for magmatic gas emissions from effusive eruptions to loft to high altitudes, even for relatively small eruptions (Boudoire et al. 2022). We compared our satellite-based results to ground-based monitoring measurements, which demonstrate the complementarity of the two approaches while highlighting the challenges of measuring a dense plume from the near-field. Lastly, we present the first PlumeTraj analysis using SO_2_ imagery from the Earth Polychromatic Imaging Camera (EPIC) onboard NOAA’s Deep Space Climate Observatory (DSCOVR). This instrument images the sunlit disk of the Earth every 1–2 h, providing multiple images per day to complement the TROPOMI results. This highlights the strength of PlumeTraj in providing a continuous time series of SO_2_ emissions from satellite SO_2_ imagery.

### Overview of Mauna Loa and the 2022 eruption

**Fig. 1:**
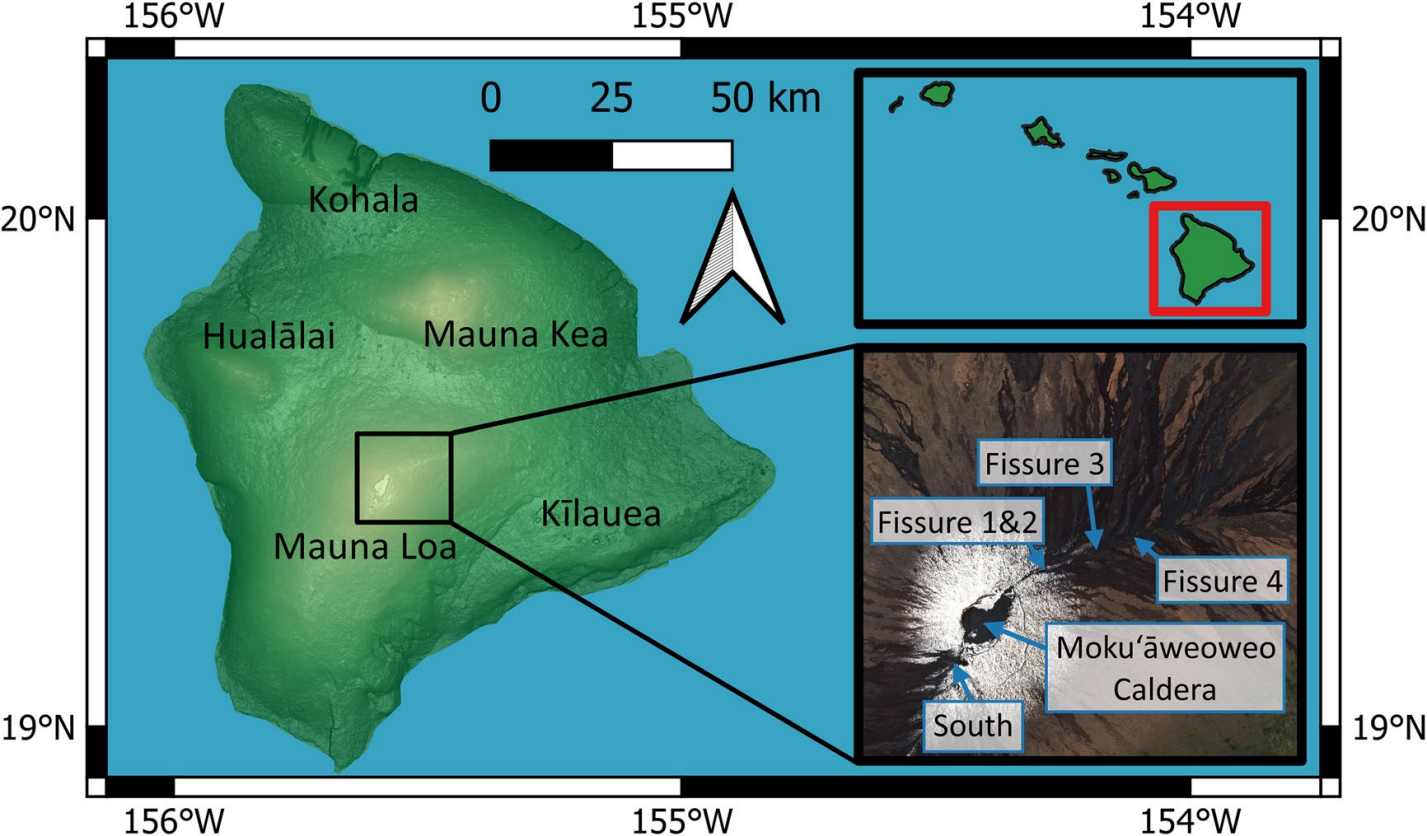
Map of Hawaiʻi, showing the locations of the volcanic centres. Inset map (top right) shows the Hawaiian Islands chain and inset image (bottom right) shows Sentinel-2 true colour image taken after the eruption (7 January 2023) with activity regions marked

Mauna Loa makes up more than half of the island of Hawaiʻi, itself the southernmost and largest of the Hawaiian archipelago (Fig. 1). Volcanic activity in Hawaiʻi is a result of hotspot activity (Wilson 1963), with the age of activity (oldest to youngest) tracking from the north-west to the south-east. Since 1832, Mauna Loa has erupted 40 times (including the 2022 eruption), with the most recent before 2022 in 1984 (Lockwood et al. 1985; Barnard 1995).

A detailed description of the 2022 eruption of Mauna Loa is given by (Trusdell et al. 2025). A summary of the key details relevant to the degassing behaviour is provided below. The eruption began at 09:30 on 28 November 2022 (all times are in UTC, HST + 10 h) in the summit caldera (Moku‘āweoweo) with a period of intense lava fountaining. Within a few hours, the summit fissure migrated to the south-west, producing lava flows but lasting only until roughly 16:30. By 16:00 on 28 November, the activity migrated as a dyke to the north-east rift zone (NERZ). Several fissures were initially active, with lava fountaining 30–60 m high; however, the activity focused over the next days to a single location (fissure 3), which remained active until the end of the eruption. A further fissure opened to the north-east (fissure 4); however, this was only active for a short time (roughly 18:00 on 29 November to 01:00 on 2 December). The lava flows produced by this eruption did not cause any significant disruption, stopping ~ 3 km from the Daniel K. Inouye State Highway 200 (the closest main road). The eruption ended on 10 December after 13 days of activity.

The initial lava effusion rate quickly peaked around 1100 m^3^·s^−1^ in the first phase from the caldera and the southern fissures, rapidly dropping and settling around 100 m^3^·s^−1^ as activity focused on the NERZ (Dietterich et al. 2025). Tilt data showed a sharp uplift at the eruption onset, followed by a rapid drop which then stabilised to a steady decrease from 29 November. Seismic tremor also showed a spike at the eruption onset, followed by a drop, then another broader peak which stabilised on 2 December. This continued until 7 December, when the tilt turned inflationary, lava effusion dropped, fountain heights increased, and the seismic tremor became unstable until the eruption end (Dietterich et al. 2023).

## Methods

### TROPOMI

In this work, we utilise SO_2_ imagery from TROPOMI, onboard the European Space Agency’s Sentinel-5P satellite (Veefkind et al. 2012). This is a polar-orbiting satellite with a swath width of 2600 km and a spatial resolution of 5.5 × 3.5 km^2^ at nadir, providing near-global coverage daily. We use the updated Covariance Based Retrieval Algorithm product (COBRA, Theys et al. 2021), which has improved signal to noise performance over the standard differential Optical Absorption Spectroscopy (DOAS) product. TROPOMI imagery provides information on the spatial distribution and magnitude of SO_2_ column amounts in the atmosphere at the time of overpass but contains no information on SO_2_ source, plume altitude, or emission altitude and time. Several methods can constrain these parameters (Theys et al. 2013); here we use the PlumeTraj trajectory analysis toolkit (Pardini et al. 2017; Queißer et al. 2019; Burton et al. 2021; Esse et al. 2023; Hayer et al. 2023), employing a forward trajectory version of PlumeTraj, which differs from the back-trajectory mode outlined in previous papers. As this is a recent update in methodology, we outline the analysis process below. Additional description is also given by Esse et al. (2025).

## PlumeTraj

To quantitatively reconstruct volcanic SO_2_ emission history from daily SO_2_ satellite imagery, we require the plume altitude, injection altitude, and injection time for each pixel of the plume. PlumeTraj achieves this (in forward trajectory mode) by initialising trajectories from the vent location at a range of altitudes and at regular timesteps throughout the eruption. In this case, we initialised trajectories from 3 to 17 km (above sea level) in 500 m steps every hour. For each TROPOMI orbit, the trajectories were initialised for the 60 h preceding the overpass time and run to an age of 60 h, except on the first and second days, where durations of 24 and 48 h were used, respectively. The trajectories are produced using the Hybrid Single-Particle Lagrangian Integrated Trajectory (HYSPLIT) dispersal model (Stein et al. 2015), driven by the National Oceanic and Atmospheric Administration (NOAA) Global Forecast System (GFS) 0.25° global meteorological data from the National Centers for Environmental Prediction (NCEP). All pixels marked as enhanced in SO_2_ in the TROPOMI product (regardless of flagged source type) within our defined region of interest (10° N ≤ latitude ≤ 30° N, 170° W ≤ longitude ≤ 125° W) are used for analysis.

**Fig. 2:**
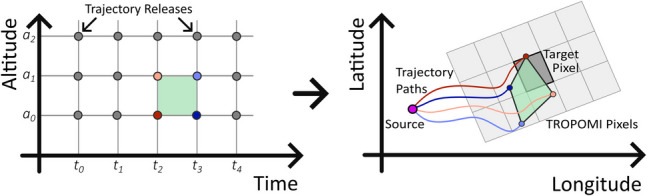
Diagram demonstrating the forward PlumeTraj method. The rectangle in altitude-time space (left) is projected onto latitude–longitude space (right), forming an irregular polygon from the trajectory positions at the time of overpass. If this polygon intersects the target pixel, then it is marked as a solution for that pixel. The process is then repeated for each set of trajectories and each analysed pixel, examining which polygons intersect it, if any

Due to natural wind shear in the atmosphere, the direction and speed of travel of the trajectories will vary with altitude, causing them to diverge downwind of the source as a function of their height. We start by defining a set of regular rectangles on a grid of time and altitude above the volcano, with the vertices defined by the trajectory release times and altitudes. We then propagate these trajectories forward onto a set of irregular polygons in latitude/longitude space, with vertices defined as the locations of four adjacent (in release time and altitude) trajectories at the time the plume was imaged by TROPOMI. Polygons which overlap with an SO_2_ pixel are flagged as a possible solution for that pixel (Fig. 2), with adjacent solutions combined to form a single solution group. In the case of more than one solution in a group, the SO_2_ mass is split between the solutions proportionally to the intersectional area between the trajectory and pixel polygons.

The introduction of four trajectories for each solution is an innovation on previous PlumeTraj approaches, which used single trajectories and their distance of closest approach to a source to calculate solutions (e.g. Pardini et al. 2017; Queißer et al. 2019; Burton et al. 2021; Esse et al. 2023). This provides greater robustness by eliminating spurious results where a single trajectory has a complex unrealistic path back to the volcano. This was particularly an issue for Mauna Loa as circulating winds in the lee of the island sometimes produced multiple solutions at similar altitudes but different injection times. Calculating the intersection of polygons is more computationally expensive than simply calculating the distance of closest approach to the source, so we adopt the forward approach as this requires significantly fewer calculations than the previous method. For example, here we generate nearly 800 trajectories per day in forwards mode (24 release times at 33 altitudes), instead of 33 times the number of pixels (up to tens of thousands depending on the plume size) in backwards mode.

Multiple solution groups can be possible for a single pixel (e.g. due to inversions in the atmosphere or changing wind conditions with time), requiring a method to choose the optimal solution. To avoid manual selection of each pixel’s solution, we select the solution with an altitude and/or time closest to a set target injection altitude and/or time (see supplementary Fig. S1). These target values are typically chosen using additional information of the plume altitude (such as from visible imagery), reported activity times (from volcanological reports) or by ensuring the results vary smoothly across the plume (e.g. minimising sudden jumps in plume altitude or age). When using both altitude and time, the altitude and time differences are scaled such that 0.5 km altitude difference and 1 h time difference are weighted equally. For this eruption, the choice of target parameters was varied throughout due to changing eruptive and meteorological conditions, with the values used given in Table 1. The choice of target parameters was varied manually to minimise discontinuities in the plume age and altitude and to maximise agreement between successive orbits. The eruption start time was used on 28 November as there were some pixels with solutions significantly earlier than the eruption start, producing a sharp jump in plume ages. Using a target injection time of 09:30 helped PlumeTraj to select the correct solutions in these cases. Using the TROPOMI overpass time was also useful in some cases for orbits where there were multiple solutions at similar altitudes but different times, caused by the swirling winds in the lee of the island. Setting the target injection time to the overpass time weights the younger solution groups more, which are assumed to have taken the shortest path from the vent to the pixel location. This was found to produce more consistent results (from orbit to orbit) than just setting a target injection altitude alone.

**Table 1:** Overview of input source locations, trajectory run times, target injection values used throughout the eruption, as well as the TROPOMI overpass times for all orbits used. Note some overpass times in UTC fall early the next day relative to local Hawaiian time

Date	Source location	Trajectory duration	Selection method (target value)	Overpass times (UTC)
28 Nov	Summit caldera (19.475° N, 155.608° W)	24 h	Injection altitude (10 km) and time (28 th Nov 09:30)	23:46
29 Nov	Fissure 3 (19.514° N, 155.530° W)	48 h	Injection altitude (6 km) and time (overpass)	21:46 and 23:28
30 Nov	Fissure 3 (19.514° N, 155.530° W)	60 h	Injection altitude (6 km)	23:09
1 Dec				22:50 and 00:30 (2 Dec)
2 Dec				00:11 (3 Dec)
3 Dec				23:53
4 Dec				23:34
5 Dec				23:15
6 Dec	Fissure 3 (19.514° N, 155.530° W)	60 h	Injection altitude (6 km) and time (overpass)	22:56 and 00:36 (7 Dec)
7 Dec				00:17 (8 Dec)
8 Dec				23:59
9 Dec				23:40

Once the plume altitude for a pixel has been quantified, we calculate the SO_2_ vertical column density (VCD). This is produced from the directly measured slant column density (SCD) using a calculated air mass factor (AMF), accounting for the observation geometry and the height dependency of TROPOMI’s sensitivity to SO_2_ in the atmosphere (with less sensitivity the lower the plume altitude). As the plume height is not known at the time of measurement, TROPOMI level 2 data provides three AMFs for 1 km thick box profiles at 0–1 km agl, 7 km asl, and 15 km asl (Theys et al. 2017). We linearly interpolate between these to the plume altitude from PlumeTraj to produce a corrected VCD, converting to SO_2_ mass by multiplying by the pixel area. With the SO_2_ mass, injection time, and injection altitude for each pixel, the SO_2_ emission time series can then be reconstructed. One of the key strengths of PlumeTraj is that a single daily TROPOMI image can produce > 24 h of SO_2_ emission data, allowing both a continuous record of gas dynamics to be reconstructed and independent inter-day comparisons to be performed.

PlumeTraj calculates uncertainties in the plume altitude from the standard deviation of the altitudes of solutions within a single solution group (with a minimum of 500 m, reflecting the grid spacing). The plume altitude uncertainty is then propagated with the uncertainties in the SO_2_ SCD and AMFs provided in the TROPOMI SO_2_ product to give the uncertainty on the final VCD and SO_2_ mass. Finally, the mass uncertainty for each pixel is combined to determine the total uncertainty in the SO_2_ emission rate.

## PlumeTraj Limitations

PlumeTraj as a tool is not perfect and there are areas in which it may not perform as well. This section will give a brief overview of the model’s limitations and what could be done in the future to address them.

1. Interpolation of air mass factors: PlumeTraj calculates an altitude-corrected AMF to account for the height dependency of the SO_2_ sensitivity. However, the simple linear interpolation between the three provided AMFs at 0.5 km, 7 km, and 15 km will lead to errors in the resulting AMF compared to if this was recalculated fully. However, the information required to fully reconstruct the AMF for a given SO_2_ profile is not included in the TROPOMI product files, meaning that we would need to incorporate the weighting function lookup table from the operational processor (Theys et al. 2017) into the PlumeTraj workflow. Implementing this is planned for future development of PlumeTraj, but this is not currently available.
2. SO_2_ loss: Currently, there is no consideration of SO_2_ loss included in PlumeTraj. After emission, SO_2_ will be lost through oxidation to SO_4_^2−^ and through wet and dry deposition, with a (tropospheric) lifetime of ~ 15 to 65 h, depending on the atmospheric conditions and injection altitude (Lee et al. 2011; Hardacre et al. 2021). Additionally, as the SO_2_ plume disperses downwind, it will drop below the satellite detection limit, further decreasing the apparent lifetime. In the future, including SO_2_ loss terms explicitly in the dispersal model could improve the overall accuracy, either using a single loss rate or by including the loss calculation within the dispersal model used to generate the trajectories.
3. Global meteorological data: Currently PlumeTraj uses the global GFS meteorological data to calculate the trajectories used. However, this data has a relatively coarse resolution (0.25°) and may not fully resolve terrain features. This could be improved in the future through the use of local mesoscale models to improve the resolution.
4. Single plume altitude: within the PlumeTraj method, the SO_2_ is assumed to be contained within a 1 km thick box profile at a given altitude (with an uncertainty). In cases where there are two layers of plume at different altitudes, this will lead to some error in the calculated SO_2_ mass. There is no easy way to address this issue, as the satellite measures the integrated column of SO_2_; however, it would be possible to investigate using an inverse modelling approach.

## DSCOVR/EPIC

To provide additional details on the eruption start, PlumeTraj was also applied to SO_2_ imagery captured by the Earth Polychromatic Imaging Camera (EPIC) onboard NOAA’s Deep Space Climate Observatory (DSCOVR). Launched in February 2015, DSCOVR is situated roughly 1.5 million km from Earth at the Earth-Sun Lagrange-1 point, allowing EPIC to image the full sun-illuminated disk of the Earth. EPIC consists of a 2048 × 2048 pixel charged-coupled device that is sensitive to radiation at UV, visible and near-infrared wavelengths, with 10 narrow-band channels on a filter wheel, covering wavelengths from 317.5 nm to 779.5 nm. EPIC images the Earth with a spatial resolution of 18 × 18 km at nadir (after down sampling to 1024 × 1024 pixels to reduce transmission time) and at a cadence of 68–110 min, depending on the season (Marshak et al. 2018). Various products are available, including for ozone, aerosols, cloud, vegetation and, importantly for volcanic applications, SO_2_ (Carn et al. 2018; Herman et al. 2018).

The SO_2_ product from EPIC is less sensitive than that of TROPOMI due to the much further distance from the Earth, the coarser spatial resolution and the multispectral (rather than hyperspectral) nature of the sensor. There is also only a single SO_2_ product available (calculated for a plume height of 13 km asl), with no reported uncertainty. Nevertheless, the sensitivity is enough to identify strong volcanic sources with columns greater than ~ 5–15 DU (Carn et al. 2018), including from the eruption of Mauna Loa.

The application of PlumeTraj to the SO_2_ imagery from EPIC differs slightly from TROPOMI. Firstly, all pixels with a reported SO_2_ VCD over 1 DU were taken for analysis. Secondly, the SO_2_ product does not contain information on the pixel bounds, only the central latitude and longitude. Therefore, instead of using pixel polygons to determine possible solutions within PlumeTraj, we used points. This does not significantly impact the results; it only means that the SO_2_ mass for a solution group is split evenly across that group instead of scaling by the relative intersectional areas. Lastly, the fact that there is only a single SO_2_ product means that no altitude correction was applied, rather we simply used this SO_2_ column (and corresponding SO_2_ mass) when calculating the emissions. This means that there is some uncertainty on the magnitude of the emissions; however, the injection altitudes and times will still be accurate.

## Results

TROPOMI provided daily images of the Mauna Loa SO_2_ plume during the eruption. Gas emissions dispersed to the east in the first two days before swinging around to the south and then west. The wind speeds significantly slowed, dropping from ~ 12 m·s^−1^ to ~ 3 m·s^−1^ (at 6 km asl) on 29 November (supplementary Fig. S2). This meant that SO_2_ emitted on the first day of the eruption into a strong wind circled the globe, while later emissions lingered around the volcano and remained detectable for several days after emission. On some days, the plume from previous days can be seen to be blown back over the vent (e.g. 1 and 3 December), complicating measurements of the emission rate from both ground and space.

**Fig. 3:**
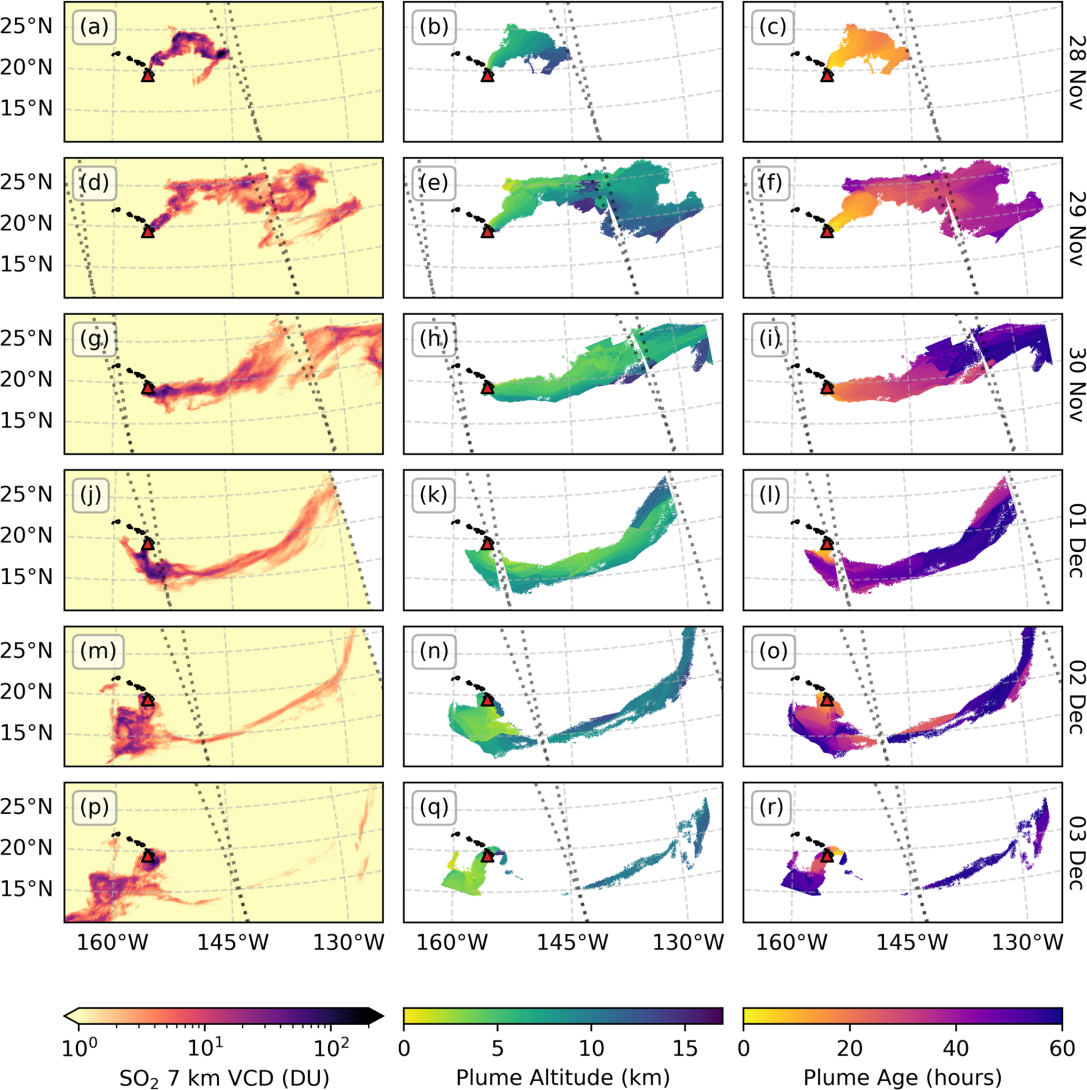
PlumeTraj results for 28 November to 3 December 2022, showing (left) the TROPOMI 7 km SO_2_ VCD product, (centre) the plume altitude and (right) plume age

**Fig. 4:**
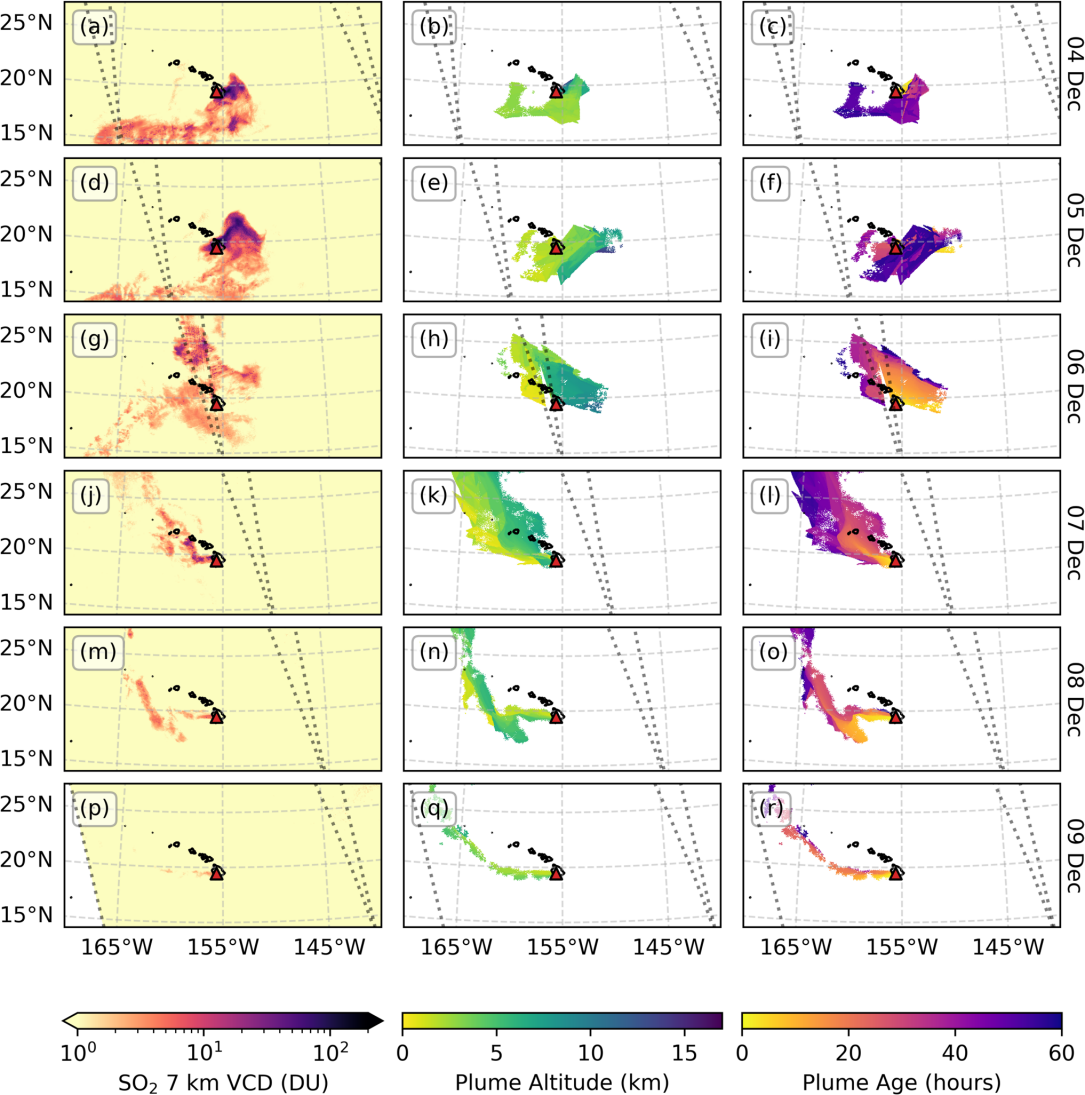
PlumeTraj results for 4 to 9 December 2022, showing (left) the TROPOMI 7 km SO_2_ VCD product, (centre) the plume altitude and (right) plume age

**Fig. 5:**
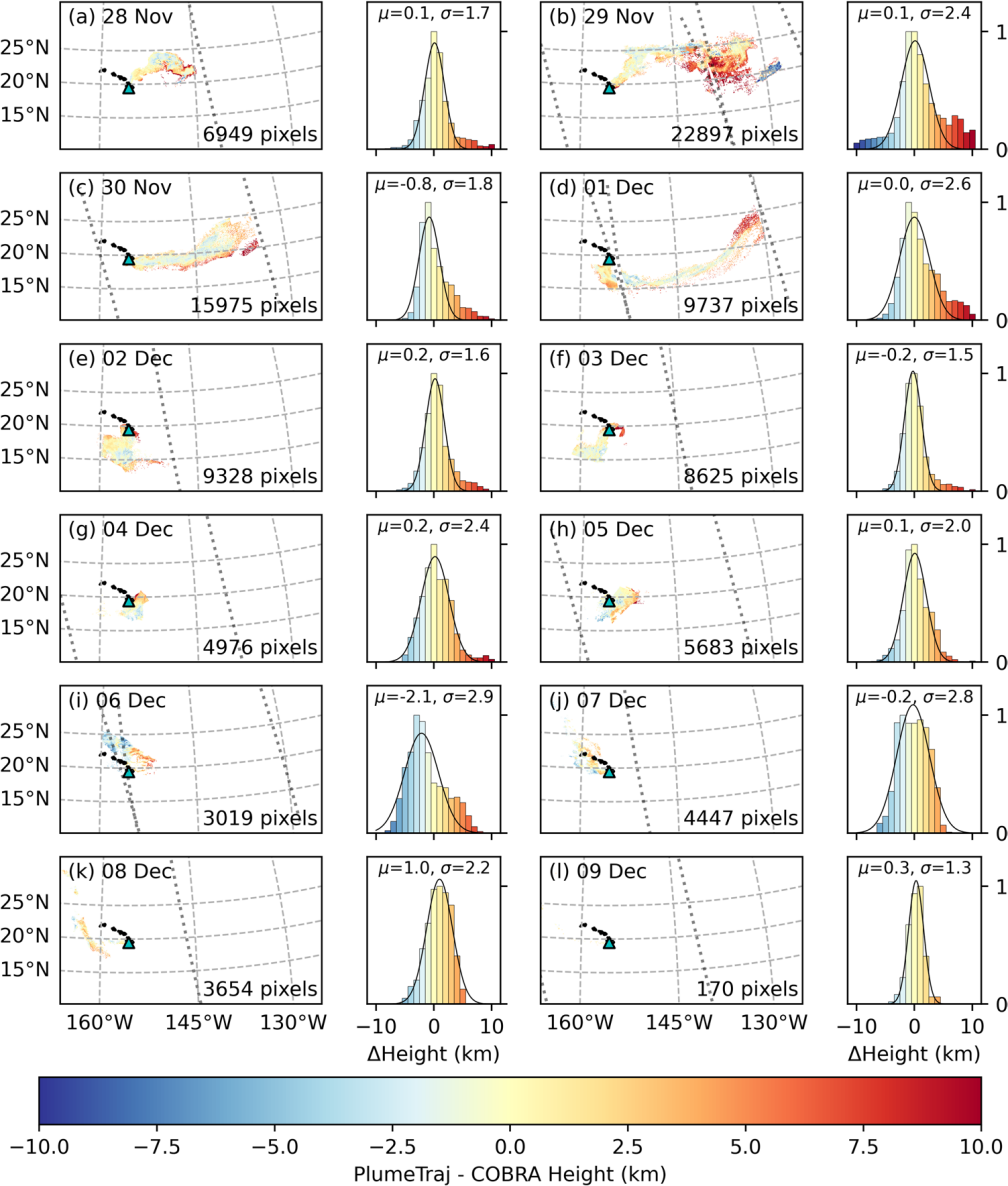
Comparison between PlumeTraj and COBRA plume heights for each day in the eruption. Maps show pixel-by-pixel difference with the swath edge given by the dotted line, while histograms give the normalised distribution in height differences in 1 km bins. Black curve gives a fitted Gaussian distribution, with the mean (*μ*) and standard deviation (*σ*) values shown (in km)

PlumeTraj was applied to each TROPOMI overpass, with results shown in Fig. 3 (28 November – 3 December) and Fig. 4 (4–9 December). SO_2_ VCDs are given in Dobson Units (DU, 1 DU = 2.69 × 10^16^ molecules·cm^−2^). Note that orbits 26623 on 2 December and 26637 on 3 December were excluded from the emission results as each contained only the old tail from previous emissions. To test how PlumeTraj performed, we compared the calculated plume altitudes with those provided by the TROPOMI Covariance Retrieval Algorithm (COBRA) layer height product (Theys et al. 2022). COBRA SO_2_ layer heights are a purely spectroscopic measurement of the altitude of the SO_2_ plume at the time of measurement, and therefore provide an independent test for PlumeTraj altitudes, which rely only on the wind field data and target parameters. The COBRA layer heights require a relatively strong SO_2_ signal, and so we filtered the data to only consider pixels with a COBRA height error of less than 2.5 km and with a corrected COBRA VCD value of greater than 5 DU. The results of this comparison are given in Fig. 5.

Overall, the agreement between PlumeTraj and COBRA is good for the first half of the eruption (until 5 December), with the observed distributions centred near 0 and with a standard deviation of ~ 2–3 km. The stronger positive skew seen on some days (e.g. 28 November – 3 December) can in part be attributed to COBRA biasing the plume altitude lower towards the plume edge where the SO_2_ signal is reducing and/or the signal is only coming from a portion of the satellite pixel. The agreement is less good in the second half of the eruption where PlumeTraj is likely struggling due to the very slow wind speeds and the changing wind direction (supplementary Fig. S2), as well as COBRA possibly struggling with lower VCDs, although the agreement improves again in the last couple of days. Comparing the model wind speeds and bearings to radiosonde data throughout the eruption (supplementary Figs. S3 and S4) shows that the agreement between the two is less good in the second half of the eruption (especially in terms of the wind direction), highlighting that the model is struggling to accurately replicate reality here.

## Eruption onset

**Fig. 6:**
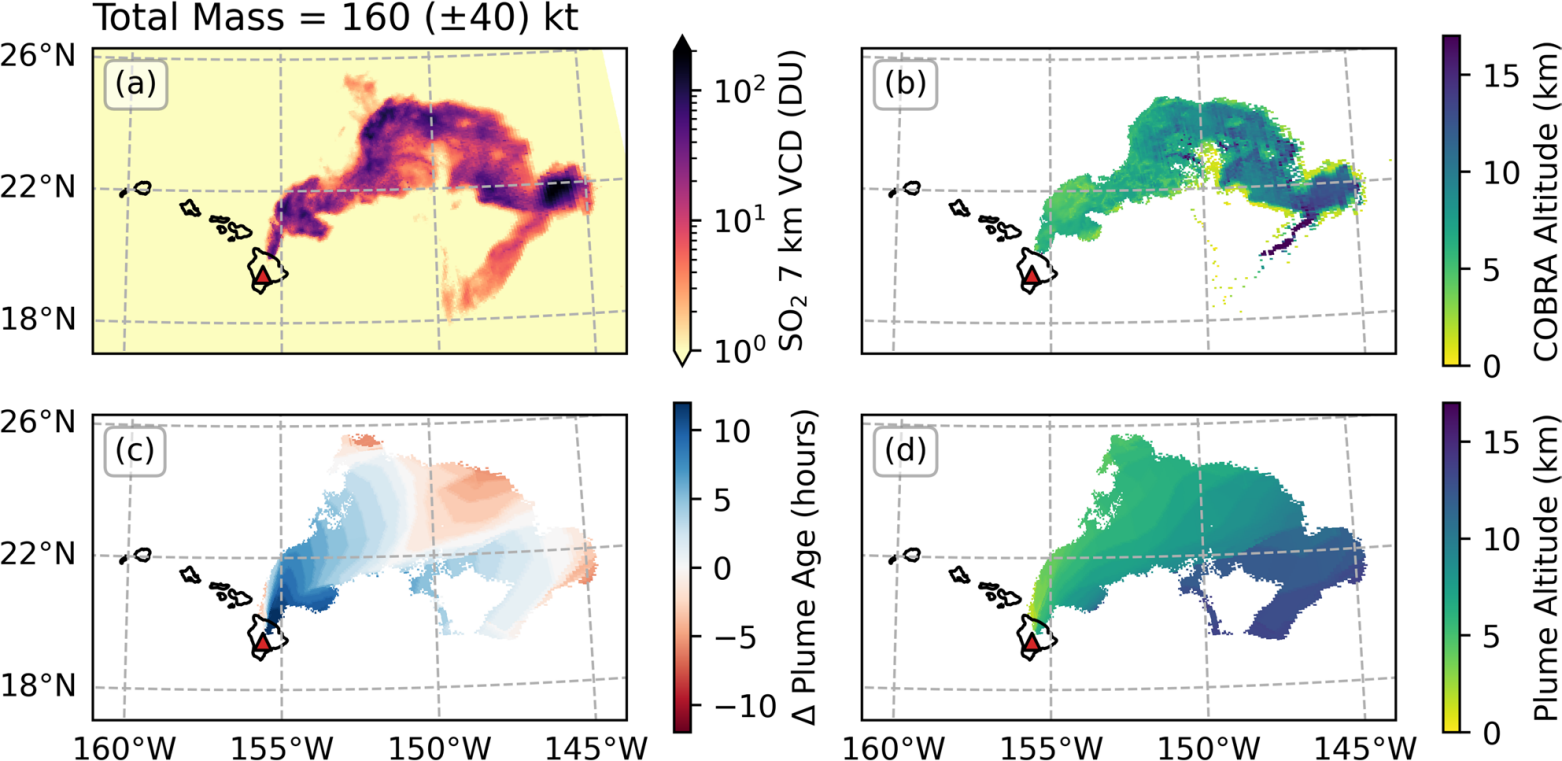
Example results from the first day of the eruption (orbit 26567). (**a**) The 7 km VCD from the TROPOMI COBRA product (log scale). (**b**) The COBRA layer height product. (**c**) The plume age difference from the eruption onset time (09:30 UTC), with red showing SO_2_ emitted before the eruption onset and blue after. (**d**) The plume altitude from PlumeTraj. The red triangle gives the vent location

The first overpass occurred approximately 15 h after the eruption onset (09:30 UTC on 28 November), capturing the initial SO_2_ emissions well. Figure 6 shows the plume age and altitude from PlumeTraj and the COBRA SO_2_ layer height, highlighting a clear high-altitude portion to the southeast of the plume.

**Fig. 7:**
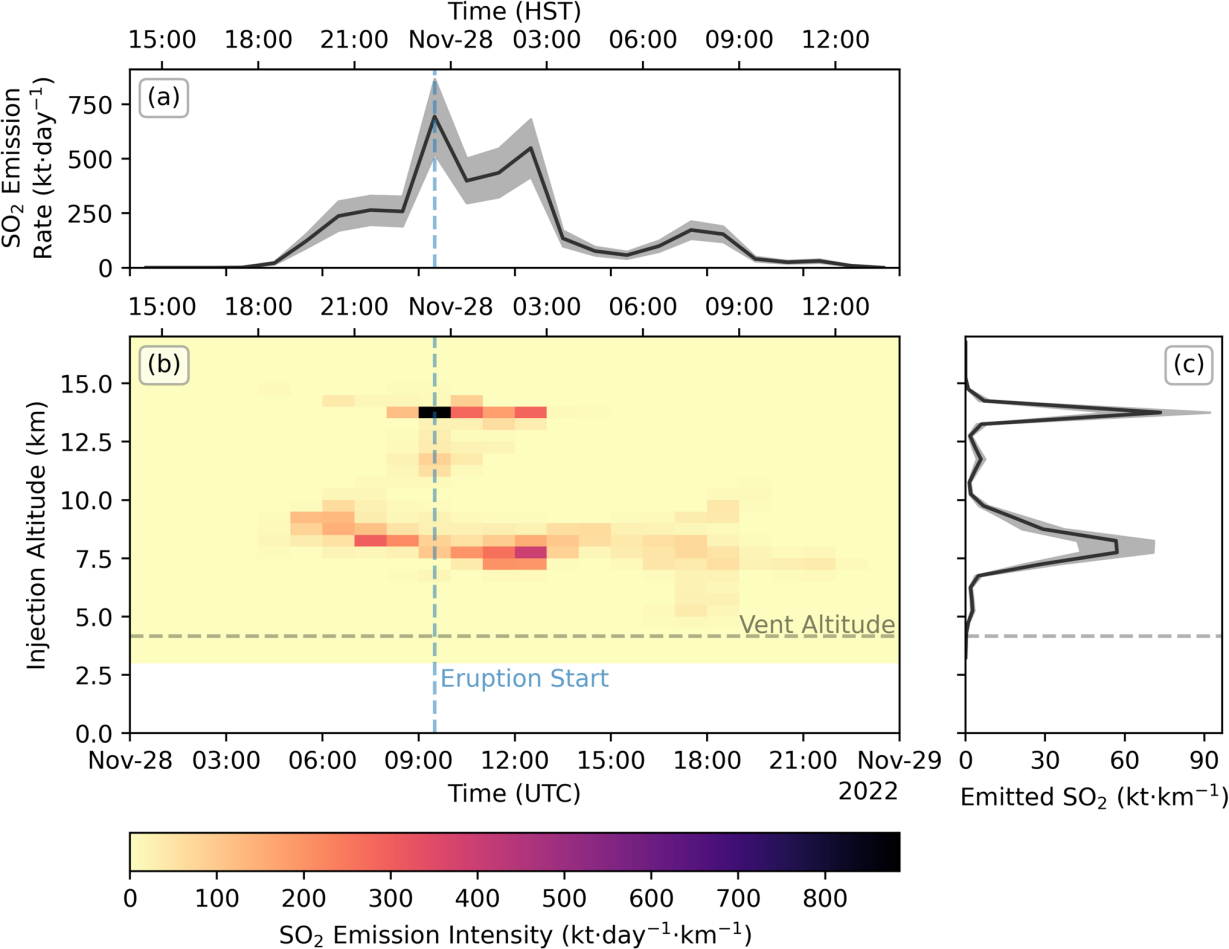
Emission results for the eruption onset. (**a**) The total emission rate, with shaded region giving calculated uncertainties. (**b**) The emission intensity as a function of time and altitude. (**c**) The total emitted SO_2_ as a function of altitude. Also given are the vent altitude (horizontal grey dashed line) and eruption start time (vertical blue dashed line)

A small portion of the south-eastern part of the plume did not successfully return due to the generated forward trajectories never reaching this section. Additionally, the western edge of the plume close to the source can be seen to have an anomalously old plume age and low plume altitude as the only trajectories reaching this edge of the plume are from a lower injection altitude with a slower speed. However, for both these cases, the SO_2_ mass in the affected regions is minor (Fig. 6a, note log scale) compared to the main bulk of the plume. The emission results are shown in Fig. 7.

There are two notable features revealed in this first image. Firstly, the PlumeTraj results show precursory emission of SO_2_ more than 3 h prior to the eruption onset at an altitude of 8–9 km. Secondly, we find a high-altitude (14 km) injection of SO_2_ coincident with the onset and cessation of lava fountaining at the summit (Pasqualon et al. 2025). This suggests the initial fountaining stage was responsible for injecting SO_2_ at altitudes approaching the tropopause height (roughly 14–16 km above sea level, determined from radiosonde data, supplementary Fig. S5) without significant explosive activity. The SO_2_ was also injected into the atmosphere at two altitudes simultaneously, suggesting that this is resolving multiple emission sources, potentially from the fountain and lava flow.

**Fig. 8:**
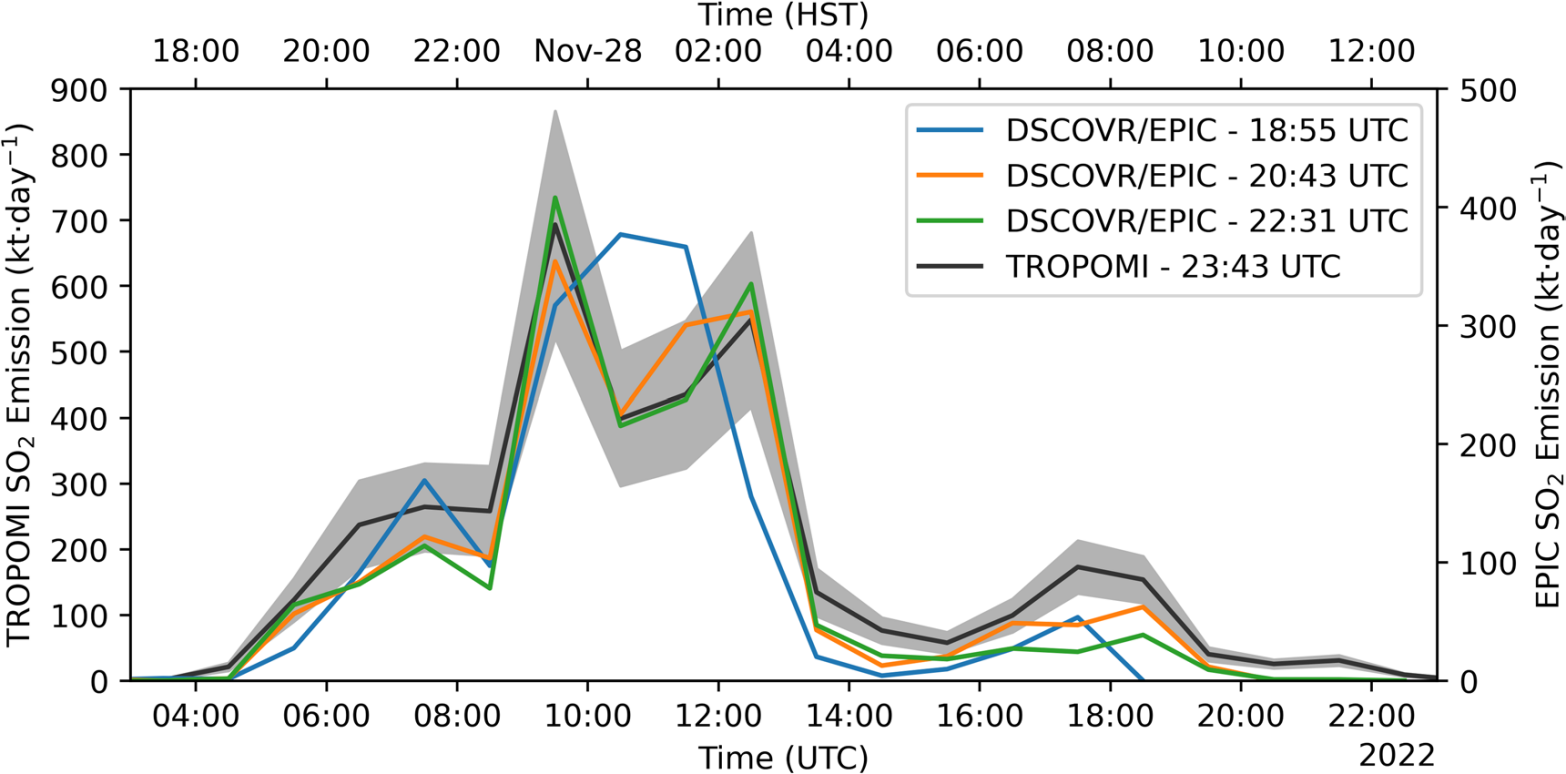
Comparison between PlumeTraj derived SO_2_ emissions on 28 November from TROPOMI (black line, shaded region showing uncertainty) with those from EPIC (blue, orange and green lines, no uncertainty calculated). Note the emissions are scaled on different axes for each instrument

To verify the timing of the precursory emissions, we also ran PlumeTraj on SO_2_ imagery from EPIC on 28 November. Three images were captured by EPIC of the plume this day, measured at 18:55, 20:43 and 22:31 (UTC), all prior to the TROPOMI measurement at ~ 23:43 (UTC). The calculated SO_2_ emission rates from EPIC are shown in Fig. 8, alongside the TROPOMI results for comparison. Plume age and altitude maps and emission profiles, are given in supplementary Fig. S6. The emissions calculated from EPIC are systematically lower than those for TROPOMI (note different scales used), likely due to the lower sensitivity and lack of altitude correction on the SO_2_ VCD. However, the emission profile shape is consistent across all four analyses, showing the precursory emissions from ~ 06:00 (UTC) and the main peak coinciding with the eruption onset.

## Full eruption

**Fig. 9:**
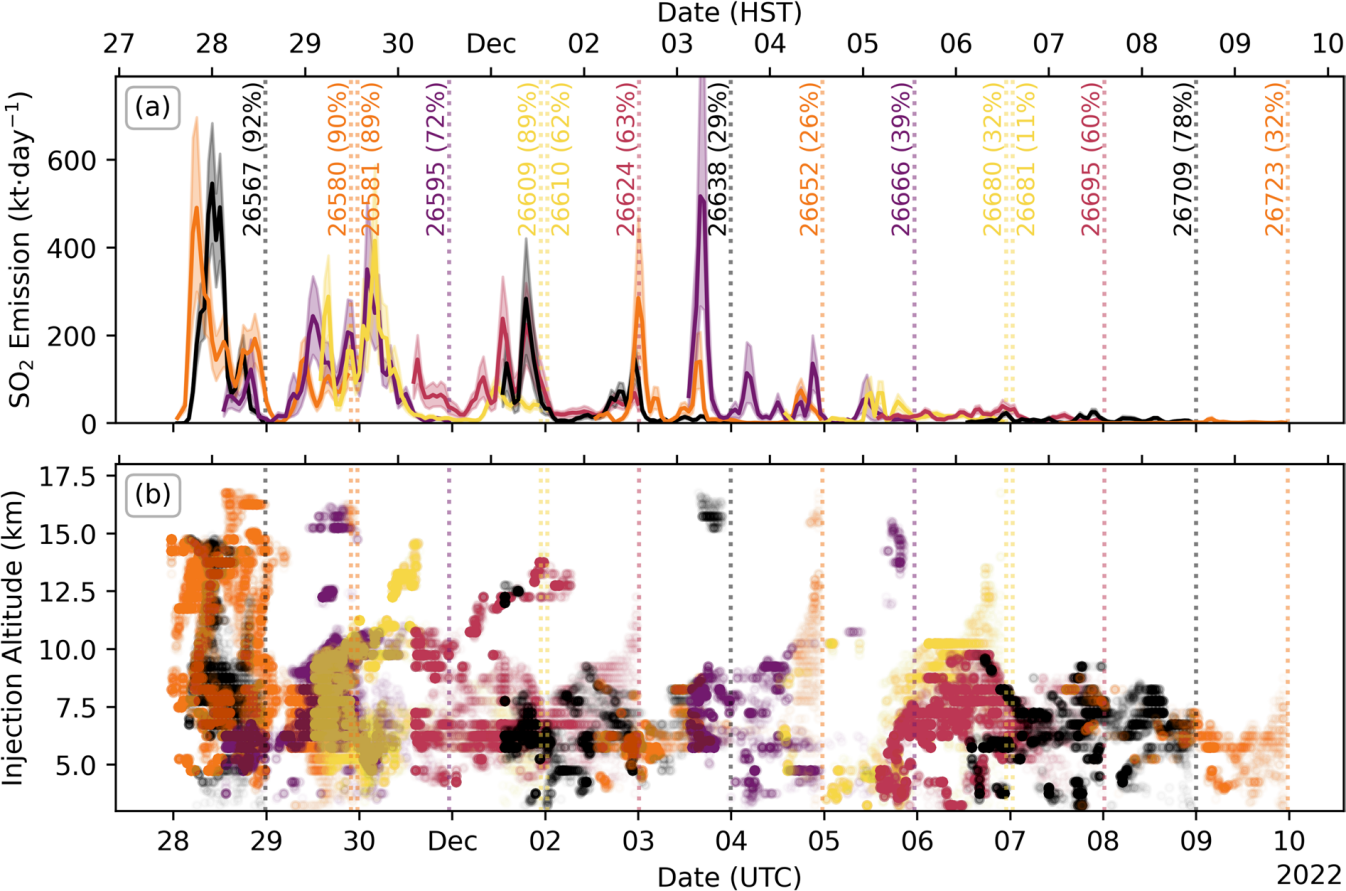
Overview of PlumeTraj results for individual orbits. (**a**) The reconstructed SO_2_ emission rate, with the shaded region giving the uncertainty calculated by PlumeTraj, and (**b**) the injection altitudes for each successful pixel return. Orbits are coloured by local measurement date, with the overpass times given by the vertical dotted line and orbit number marked. Numbers in brackets give the percentage of SO_2_ enhanced pixels that were successfully returned by PlumeTraj

The SO_2_ emission results for each TROPOMI orbit analysed with PlumeTraj, grouped by day of measurement, are shown in Fig. 9. This shows both the total SO_2_ emission rate and injection altitudes for all pixels analysed.

Two clear peaks in both emission rate and injection altitude can be seen in the first days of the eruption, one at the eruption onset and another from roughly 10 am on 29 November (UTC). These show remarkable agreement between consecutive orbits, both in terms of emission rate and injection altitude, with the timings of individual peaks conserved across up to three days of measurements. This suggests that the SO_2_ emission was highly dynamic during this initial phase, which is not reflected in the recorded seismicity, and likely reflects the evolution of the shallow fissure system as the main centre of activity shifted from the summit caldera to the northeast rift zone.

Small bunches of pixels can be seen occasionally returning at high altitude for the rest of the eruption (orbits 26638 and 26666); however, these are sourced from the plume edge and are small in SO_2_ mass, most likely due to either plume spreading or small errors in the meteorological data producing spurious solutions. Increases in injection altitude just before the overpass (orbit 26652) are caused by a lack of time for the trajectories to spread downwind, meaning that possible solutions are spread across a wide range of altitudes, which is captured in the uncertainty analysis. The timing of the drop in injection altitude seen on 9 December is coincident with the onset of fluctuations in the recorded seismic energy release, a drop in thermal radiative power and lava effusion rate, as well as an increase in the lava fountain height (Dietterich et al. 2023).

**Fig. 10:**
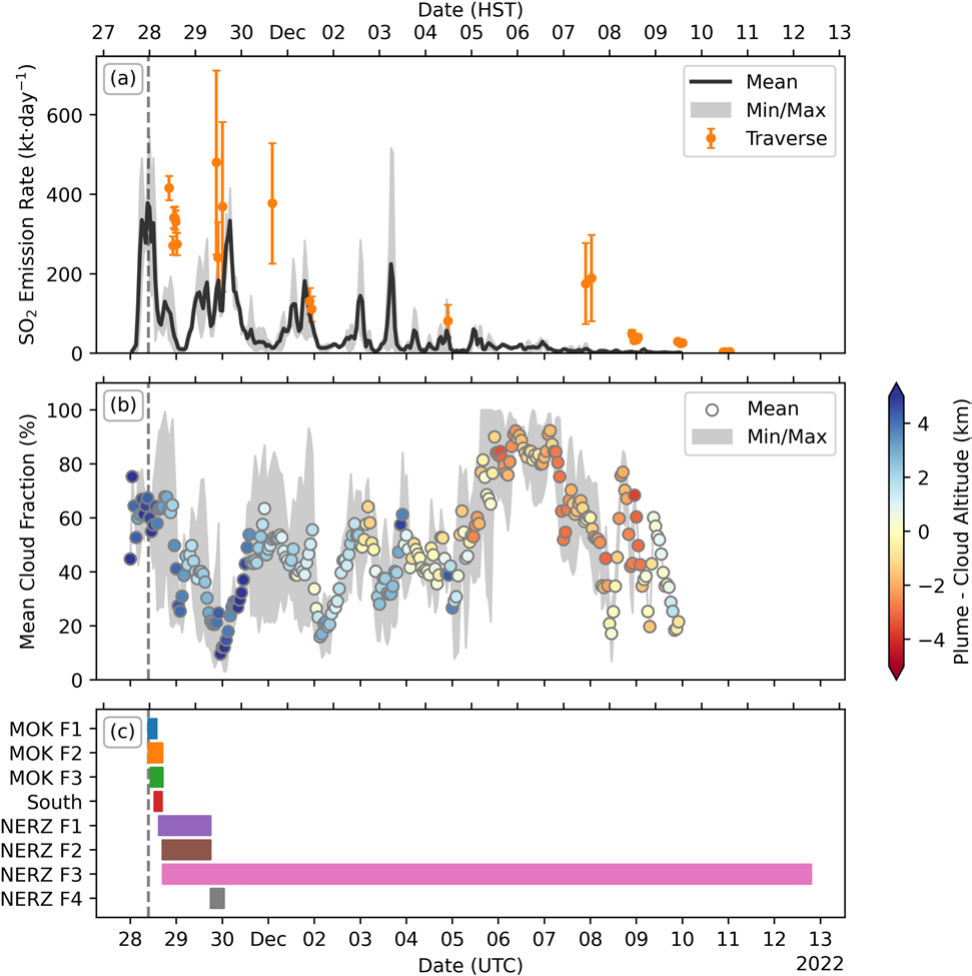
Comparison of PlumeTraj SO_2_ emission with traverses, meteorological cloud and vent activity times. (**a**) Mean, minimum and maximum SO_2_ emission rates from PlumeTraj analyses of different days (black line and shaded region) with ground-based traverse measurements (orange circles). (**b**) Mean, minimum and maximum cloud fraction time series from TROPOMI images. Points are coloured by the mean plume–cloud altitude difference. (**c**) Vent activity times from Trusdell et al. (2025), coloured by fissure number

To produce a single SO_2_ emission time series we took the mean emission rate of all orbits at each time step, as well as the minimum and maximum values, and compared this to ground-based measurements collected using the traverse method (Fig. 10a). Traverses were performed as often as possible throughout the eruption by driving a zenith pointing spectrometer underneath the plume and multiplying by the wind speed (Nadeau et al. 2023). We also calculated the mean cloud fraction of all pixels contributing to each time bin in the emission measurement, as well as the mean plume–cloud altitude difference to determine if the variations in the emission rate produced by PlumeTraj could be attributed to changes in the cloud conditions (Fig. 10b). The cloud fraction and altitude were taken from the TROPOMI cloud product (Loyola et al. 2018), calculated using the Optical Cloud Recognition Algorithm (OCRA) and available within the L2 SO_2_ product files (Romahn et al. 2022).

These results show that, although in the first half of the eruption the overall magnitude of the PlumeTraj and traverse emission rates are similar, they do not always align in time. Additionally, towards the end of the eruption the emission rates from PlumeTraj are systematically lower than the traverses. Looking at the cloud results, we can see that while there is significant cloud (~ 60%) present at the eruption onset, the plumes are at a substantially higher altitude than cloud (> 2 km) and therefore will not be obscured by the cloud. This, combined with the fact that the temporal evolution in emission rates is preserved across multiple orbits, suggests that the SO_2_ emission variation calculated by PlumeTraj is real. Towards the end of the eruption, we see that the cloud cover increases and the plume – cloud altitude difference drops, suggesting that the SO_2_ is being obscured by meteorological cloud which would result in the emission rate being underestimated.

The disagreement between the traverse and PlumeTraj emission rates on 28 and 30 November is somewhat surprising. The traverses were taken around midday local time, at roughly the same time as the TROPOMI overpasses. Close to the vent, the emissions measured by TROPOMI can be underestimated due to saturation of the absorption features and/or the optical thickness of a dense, aerosol-rich plume; however, the fact that PlumeTraj analysis of three consecutive days of TROPOMI imagery give almost identical SO_2_ emissions and the good agreement with traverses seen elsewhere in the time series would suggest that the PlumeTraj measurements are robust. On 30 November, the plume can be seen to be circling back over the summit area due to the change in wind direction, so it is possible that the traverses are being contaminated by this previously emitted SO_2_, although this would not explain the discrepancy on 28 November. We note that the agreement between the magnitude of the traverse and TROPOMI SO_2_ emissions is much closer than for the 2018 eruption of Kīlauea (Kern et al. 2020; Delbrel et al. 2024). This may be a result of the higher plume altitudes produced by Mauna Loa, which would favour TROPOMI observations due to the decreased sensitivity to SO_2_ at lower altitudes, as well as less shielding of the plume by cloud. The Mauna Loa traverse measurements were also less complicated than for Kīlauea, not requiring the same level of radiative transfer corrections due to the lower optical thickness of the plume, which may also contribute to this.

Looking at the vent activity times, we can see that the first peak in SO_2_ emission aligns with the activity at the summit caldera (MOK F3) and, as already mentioned above, the timing of the high-altitude emissions aligns well with the onset and cessation of lava fountaining at the summit (Pasqualon et al. 2025). The onset of activity at fissures 2 and 3 is during the waning phase of the initial peak, with no increase in the SO_2_ emission seen until after 06:00 on 29 November (UTC). This is in rough agreement with the observed increasing trend in lava fountain height, which peaks on 29 November (although determination of the exact timing was hampered by poor visibility (Trusdell et al. 2025). The sub peak at around midnight on the morning of 30 November is roughly aligned with the activity at fissure 4.

**Fig. 11:**
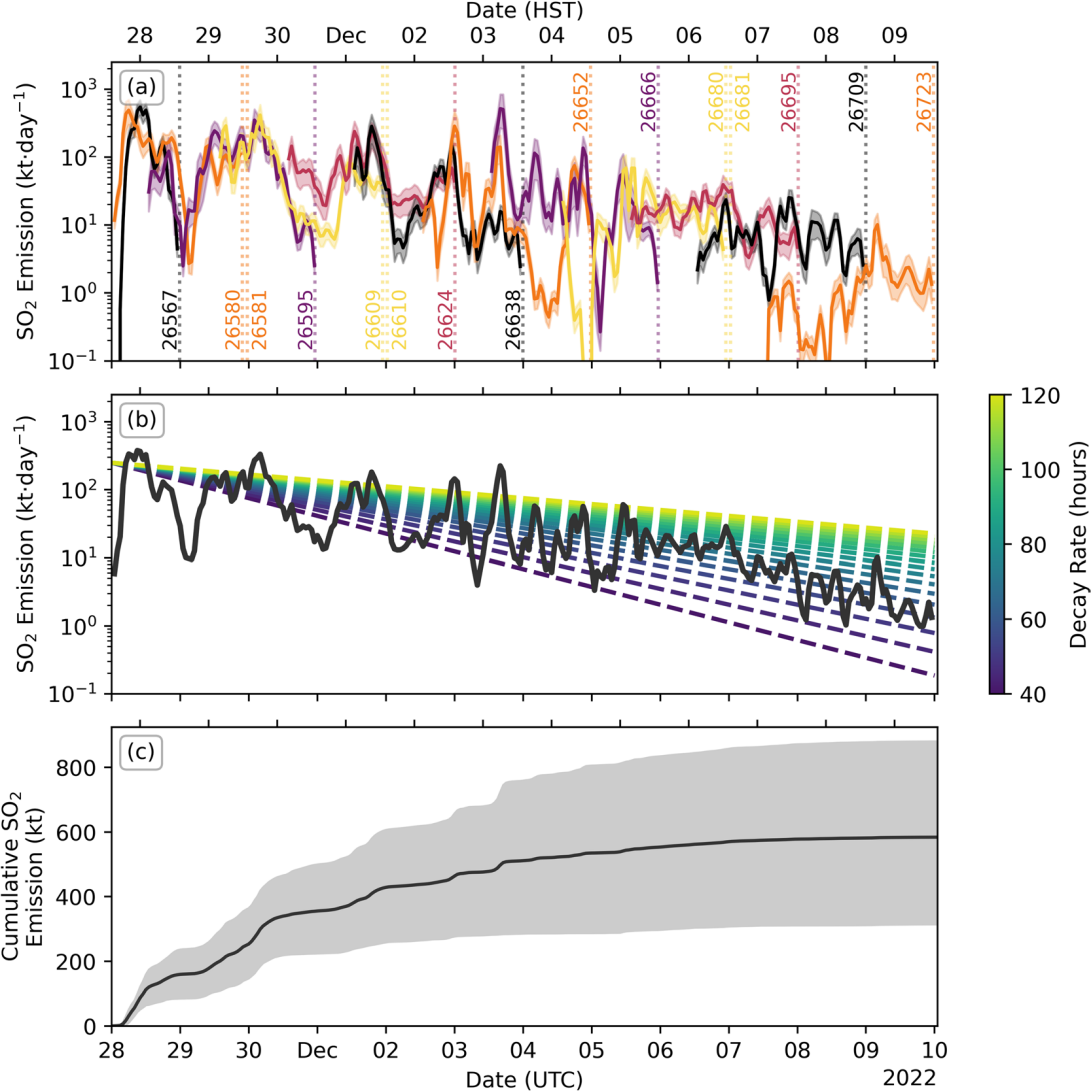
Overview of emission results in log space for (**a**) orbit-specific emissions and (**b**) mean emissions (solid black line), with modelled decays shown (coloured dashed lines), and (**c**) the cumulative SO_2_ emission throughout the eruption. Grey shaded region gives the minimum and maximum values (from Fig. 10a)

The SO_2_ emissions show strong variability, but there is an overall decay in the emission rate throughout the eruption. Plotting the emission results from Figs. 9 and 10 in log space (Fig. 11), we see a roughly linear drop (exponential decay) in the emission rate with time. Modelling a simple exponential decay starting at 250 kt·day^−1^ shows that a decay rate of roughly 50–100 h would fit the observed gradient (Fig. 11b). Plotting in log space also highlights the good agreement between consecutive orbits in the first days of the eruption, which breaks down in the later days as the accuracy of the meteorological data decreases. This provides a useful self-consistency check when analysing satellite-derived emission rates with PlumeTraj. The total emitted SO_2_ calculated by PlumeTraj for this eruption is 600 (± 300) kt.

## Discussion

The reconstructed SO_2_ emissions throughout the 2022 eruption highlight the potential of high time-resolution gas data to better understand eruption mechanisms. These results show several interesting features.

## Precursory emissions

The reconstructed emissions show precursory degassing of SO_2_ more than 3 h before the onset of lava fountaining at the summit. This is an unexpected result and, if confirmed, may open new strategies for eruption monitoring at Mauna Loa. We therefore examined this observation carefully to see if there was any other plausible explanation. Comparing the plume ages to the eruption start time, we can see that this precursory degassing comes mainly from the north-east part of the plume (Fig. 6c). The contributing pixels for the most part have a single solution and are not confined to the plume edge. This means that this emission is not a result of the choice of target parameters, nor could it be produced by an umbrella cloud or other process pushing the plume edges against the wind field.

**Fig. 12:**
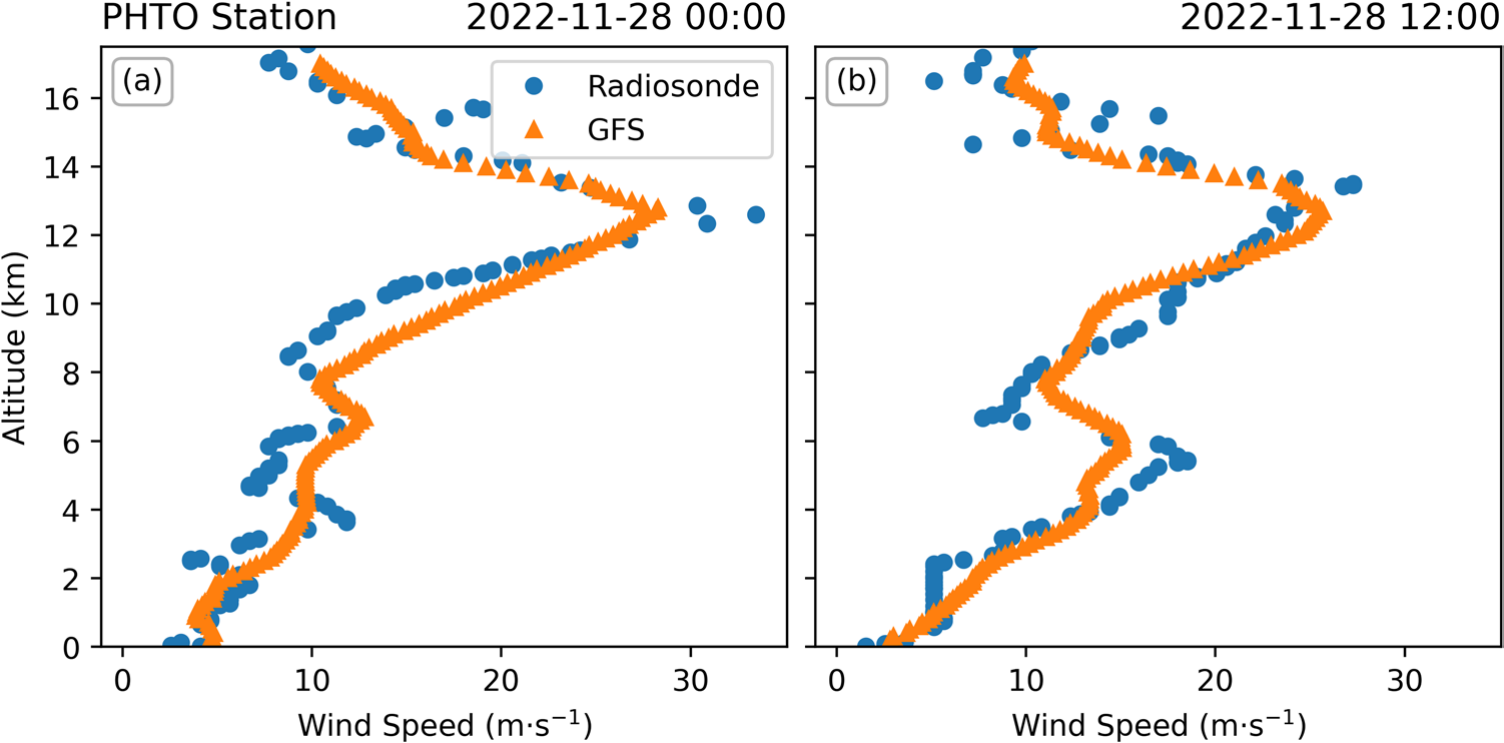
Comparison of wind speeds measured by radiosonde launches from the PHTO station (19.72° N, 155.05° W, blue circles) and from the GFS meteorological data taken above the summit (orange triangles) for (**a**) 00:00 and (**b**) 12:00 (UTC) on 28 November

There are two possible explanations for this measurement. Firstly, it could be due to errors in the trajectory analysis. The magnitude of the offset (> 3 h) is larger than the typical PlumeTraj uncertainty in plume age (< 1 h in this case), ruling out random errors in the wind fields. Alternatively, there could be some systematic offset in the wind fields that resulted in these trajectories taking too long to reach this region by the time of measurement. However, we suggest that this is unlikely as the timing (both beginning and end) of the high-altitude emissions aligns with the lava fountaining at the summit, suggesting that the meteorological data is accurate. This is reinforced by comparing modelled wind speeds for the two radiosonde launches from the PHTO station (19.72° N, 155.05° W) straddling the eruption onset, showing similar speeds (Fig. 12). There is a difference in speeds at around 8–11 km asl shown in the midnight radiosonde release (Fig. 12a); however, here the model wind speeds are faster than the measured ones, which would result in a later emission in the PlumeTraj results, not an earlier one. Finally, the emissions calculated from DSCOVR/EPIC (Fig. 8) show the precursory degassing consistently across the measurements spanning ~ 5 h. If there was a systematic offset in wind speed, then we would expect to see a drift in the timings of the emissions, but this is not the case.

**Fig. 13:**
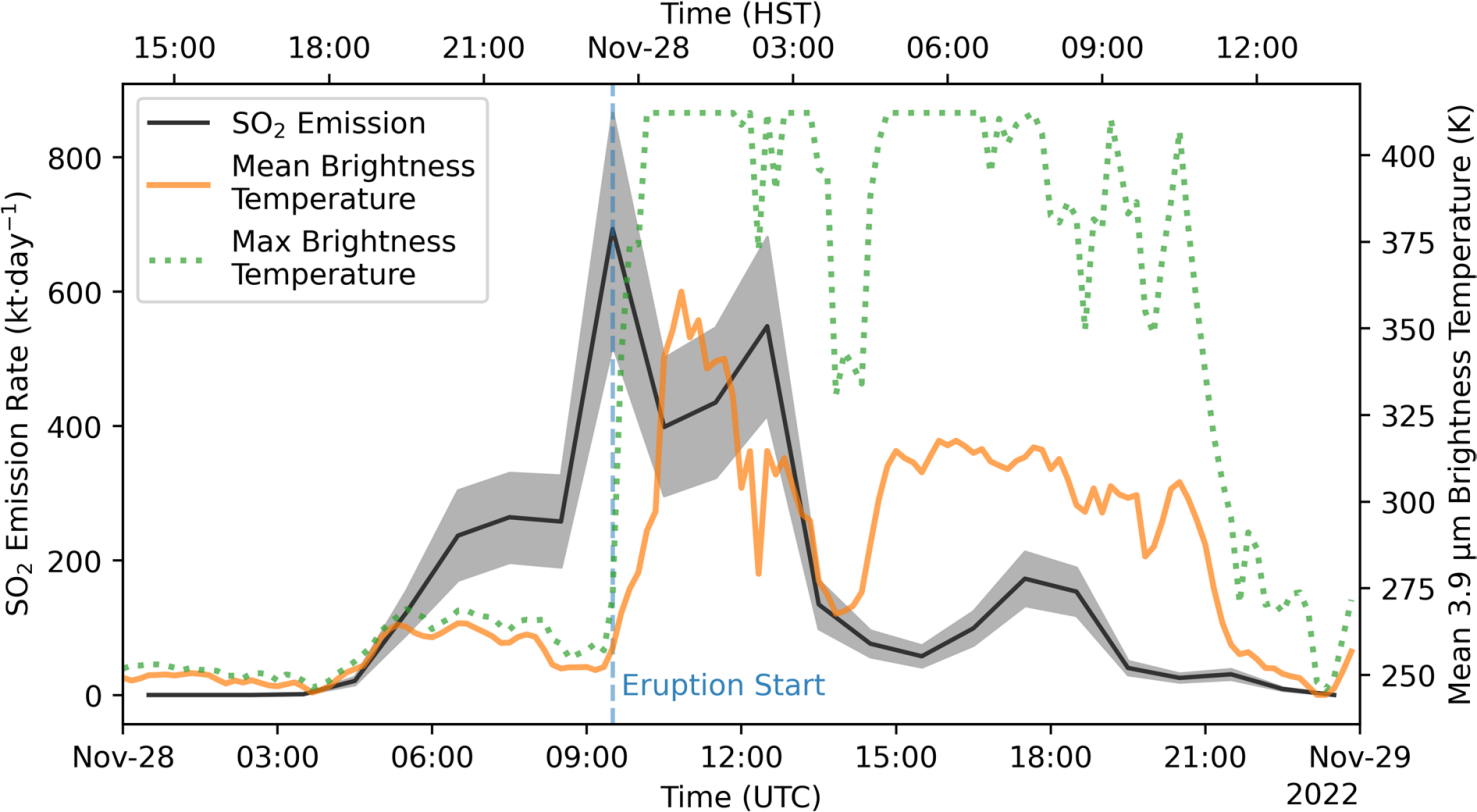
Comparison between PlumeTraj-derived SO_2_ emission rate (black line, shaded region gives uncertainty) with 10-min resolution 3.9 µm mean and maximum brightness temperatures from ABI/GOES-17 for a 10 km radius about the summit of Mauna Loa (orange solid and green dotted lines, respectively). Note the sensor saturates at 412 K. Vertical blue dashed line gives the reported eruption start time

The second option is that these are real precursory emissions that were not identified at the time, likely due to poor weather conditions at the summit and the fact that the eruption started at night. Comparing the PlumeTraj-derived SO_2_ emissions to the shortwave infrared (3.9 µm) brightness temperature from the Advanced Baseline Imager (ABI) onboard the Geostationary Operational Environmental Satellite (GOES-17) (Fig. 13) shows a clear increase in thermal emission at the eruption start, but also another, smaller increase that is coincident with the observed onset of precursory degassing. There was a MultiGAS station in the summit caldera which detected no SO_2_ prior to the eruption; however, the last SO_2_ measurement was at 18:00–18:30 (HST), just before the onset of precursory degassing calculated by PlumeTraj (Kelly, personal communication). This station was destroyed by the lava flow in the summit at the eruption start. The high solubility of sulphur in Hawaiian magmas means that sulphur would likely not exsolve until depths of 100–200 m (Lerner et al. 2021). This would mean that magma would have to ascend to very shallow depths to allow a measurable flux of gas and heat to the surface. Two magma storage depths have been identified at Mauna Loa from earthquake locations and fluid inclusion barometry at depths of 1–2 km (upper reservoir) and 3–5 km (intermediate reservoir). The triggering of the eruption is attributed to the transfer of magma into the upper reservoir, producing sufficient overpressure to exceed the point of failure (Lynn et al. 2024), and so it is possible that there was magma resident at depths shallow enough to exsolve sulphur shortly before the eruption began.

This observation suggests that near real-time measurements may have the potential to detect precursory gas emissions, which would herald the onset of an eruption. As the polar orbiting TROPOMI instrument only provides one image per day, there would need to be a fortuitous timing to capture and identify the precursory gas emission before the eruption began. However, the new generation of geostationary UV satellites, including TEMPO, GEMS, and Sentinel-4, as well as other platforms such as DSCOVR/EPIC, may offer the opportunity for such observations for Hawai‘i and at other volcanoes around the globe, supplementing existing broadband infrared geostationary satellites, such as GOES.

## Variability in SO_2_ emissions

There were two main pulses in SO_2_ emission seen in the first days of the eruption, linked to the onset of the eruption at the summit and the activation of fissures 3 and 4. Importantly, this observation was missed by traverse measurements due to the low temporal coverage, particularly through the night when traverses are not possible due to using scattered sunlight as the light source. It is perhaps surprising to have such large variations in the measured SO_2_ emission without similar variation in the lava effusion rate (Dietterich et al. 2025), especially given the high solubility of sulphur in Hawaiian magmas (Lerner et al. 2021). The agreement between multiple TROPOMI orbits suggests that this is a robust measurement and not due to an error in the wind fields. We can also rule out shielding of the plume by cloud, again due to the agreement across multiple orbits and the fact that the clouds present were at a much lower altitude than the measured plume (Fig. 10b). This leaves two hypotheses: either (1) the SO_2_ emission rate was constant and the SO_2_ was removed from the atmosphere post-emission, or (2) the magma supplying the activity had a variable sulphur content.

For hypothesis 1, there were significant clouds present during the first days of the eruption, and the weather was reportedly poor, and so it is possible that SO_2_ was efficiently removed from the plume through wet deposition. This process involves dissolving the SO_2_ in aqueous water droplets which are then gravitationally deposited (e.g. as rain). As the satellite measures this portion of the plume 1–3 days after emission, the SO_2_ would be removed before being measured, producing a consistent gap in emissions across the orbits. Assuming the emission was constant across this gap for ~ 14 h at ~ 100 kt·day^−1^, this would mean a scrubbed mass of up to approximately 60 kt.

For hypothesis two, if the SO_2_ emission was truly varying, then this would instead suggest a change in the sulphur content of the magma erupted. Looking at the vent activity times (Fig. 10c) the dip in emissions is roughly aligned with the cessation in activity at the summit and the onset of activity in the NERZ, with the second peak beginning ~ 18 h after the onset of fountaining in the NERZ. It is conceivable that to erupt lava from the NERZ would require first the extrusion of older degassed magma with a lower S-content, perhaps left behind in the dike from the 1984 eruption (Amelung et al. 2007). The uptick in SO_2_ emission would then represent the arrival of the fresher, S-rich magma. The distance from the summit to fissure 3 is ~ 8.5 km, which would mean a lateral transport speed of a little over 10 km·day^−1^. Further supporting this hypothesis is the fact that the lava fountain heights during the dip in emissions are lower (10–40 m) than in the following peak (50–80 m) (Dietterich et al. 2023), which could be explained by a lower volatile content of the magma.

## Controls on SO_2_ injection altitude

We see a strong variation in the altitude of the plume throughout the eruption, with the initial phase almost reaching the tropopause, the second peak reaching around 10 km asl and the later emissions remaining around 6 km asl. Although the lava effusion rate in the initial phase of the eruption is high (~ 1100 m^3^·s^−1^), it quickly drops to approximately 100 m^3^·s^−1^ and remains roughly constant until the eruption ends (Dietterich et al. 2025). This suggests that the magma supply rate is not controlling the injection altitudes.

Unlike explosive eruptions (Sparks 1986; Aubry et al. 2021), there is no empirical relationship between the mass eruption rate and the plume altitude from fissure eruptions due to the lack of significant ash in the plume to supply the required heat to keep the plume buoyant. Fissure plumes are controlled more by the local meteorological conditions, especially the moisture content of the ambient air (Woods 1993a, b), and (for larger scale eruptions) from heat emitted by emplaced lava (Kaminski et al. 2011). This produces a wide range of potential plume heights for similar eruptive styles and mass eruption rates.

**Fig. 14:**
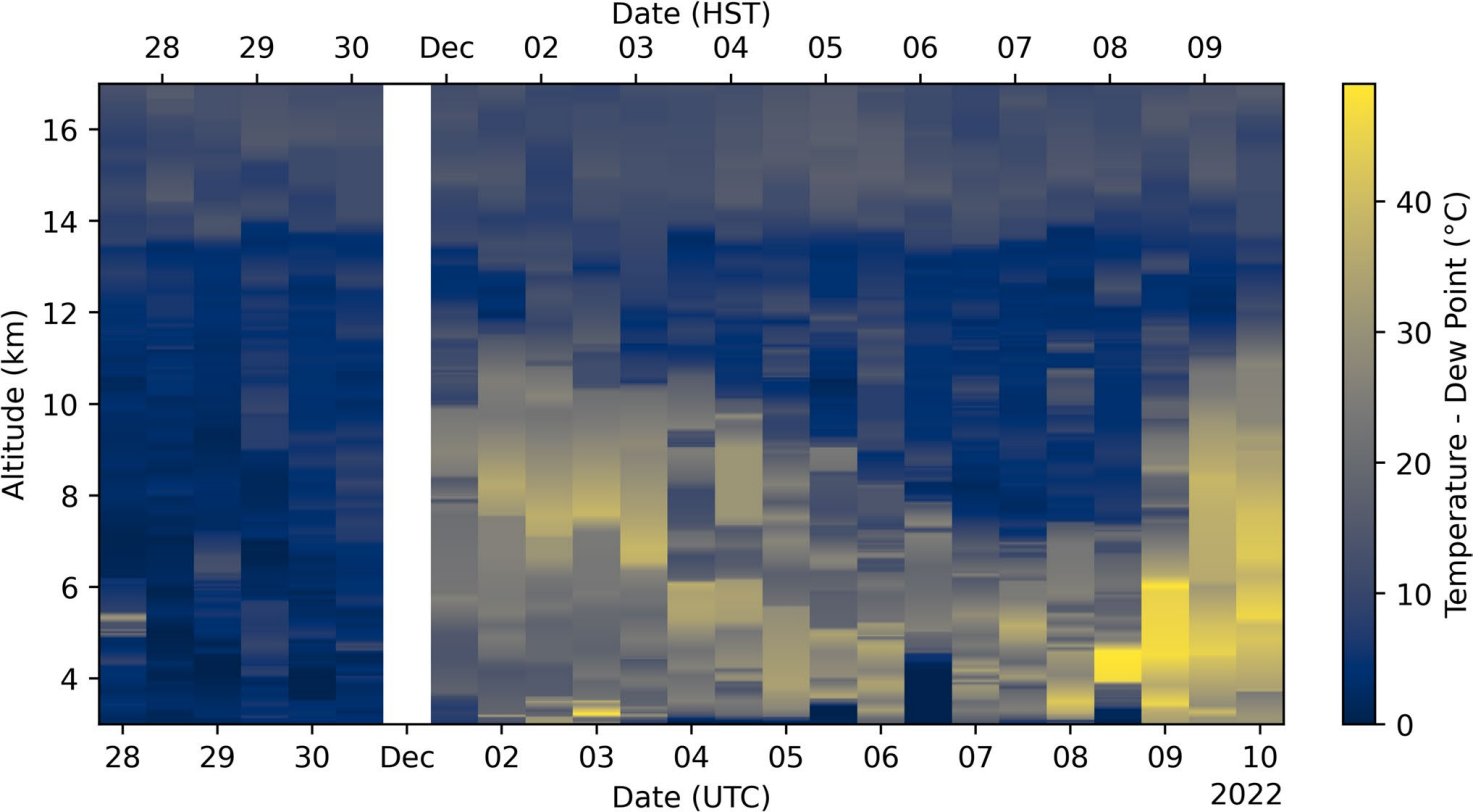
Difference in air and dew point temperature from radiosonde launches from the PHTO station (19.72° N, 155.05° W) throughout the eruption. Launches were twice a day, except on 1 December, where one launch failed. A lower difference represents a higher moisture content

Radiosonde launch data from the PHTO station (19.72° N, 155.05° W) shows that there was more available moisture at eruption altitudes in November than in December (Fig. 14), providing a driver to achieve higher altitude injections of SO_2_. We express this in terms of the difference between the air and dew point temperatures, with a lower difference indicating a higher moisture content, to remove the altitude dependence on relative humidity. This could also help to explain how the precursory emissions could reach 4–5 km above Mauna Loa. Entrainment of ambient air will rapidly cool any hot gas emissions, but the available moisture could have facilitated this rise.

Additionally, the fissure orientation at the summit was roughly NE – SW (Fig. 1), which aligns with the wind direction at the time of the eruption onset (from the south-west). This means that the wind will blow the emitted gas over the hot fissure, which itself is emitting lava and gas so that the plume is forced to rise. Although this could not explain reaching the tropopause (due to rapid cooling by entrained air), it could perhaps kick-start the rise of the plume, triggering later ascent driven by the condensation of entrained water. This requires further investigation but could have significant implications for the volcanic gas hazards from fissure eruptions (and related uncertainty) simply due to the orientation of the local wind and fissure geometry and availability of ambient water. This could also play a role in the climate impact potential of larger scale fissure eruptions.

## Conclusion

In this paper, we present SO_2_ emissions from the 2022 eruption of Mauna Loa, derived using the PlumeTraj trajectory analysis toolkit and daily TROPOMI SO_2_ imagery. These results highlight that PlumeTraj/TROPOMI can self-consistently reconstruct the SO_2_ emission time series, showing good agreement between consecutive days of measurement. We also present the first PlumeTraj analysis using SO_2_ imagery from EPIC, which further support the TROPOMI observations.

The first half of the eruption showed several interesting characteristics: there were precursory SO_2_ emissions detected over 3 h before the reported eruption start, the initial phase of the eruption injected SO_2_ at altitudes approaching the tropopause, and significant variation in the magnitude of emissions was observed. Analysis of the second half of the eruption was hampered by complex weather patterns (namely a rotation in wind direction coinciding with a reduction in wind speed) and increasing cloud cover obscuring the plume. However, a roughly exponential decay in the SO_2_ emissions as the eruption waned can still be seen, suggesting that the activity was primarily driven by the draining of a single reservoir of magma (Wadge 1981).

We highlight that accurate quantification of the emission rate and injection altitude of SO_2_ is key as these strongly affect the gas dispersal. Although the 2022 eruption of Mauna Loa was not large enough to significantly impact climate, the ability of larger fissure eruptions to inject significant quantities of SO_2_ into the upper troposphere or stratosphere would greatly increase their climate-altering potential. Conversely, gas emissions that remain near to the ground are hazardous through degradation of air quality, so understanding the mechanisms under which this does or does not occur is vital. The high-altitude emissions from Mauna Loa were likely driven primarily by the high moisture content in the ambient air in the initial phases of the eruption, with alignment of the vent geometry and wind direction also possibly providing a mechanism to initially loft the plume. Later emissions were likely constrained to lower altitudes due to a lower moisture content in the atmosphere. This demonstrates how fissure eruptions can display highly dynamic eruptive features dependent on multiple factors, making forecasts of the plume dispersal particularly difficult. The combination of PlumeTraj and TROPOMI could be used in the future to provide the SO_2_ injection source term in near real-time, helping to address this issue.

## Supplementary Information

Below is the link to the electronic supplementary material.

Supplementary file 1 (PDF 1.25 MB)

## Data Availability

The TROPOMI COBRA SO_2_ data used in this study can be accessed from the Sentinel-5P Product Algorithm Laboratory (https://data-portal.s5p-pal.com/). The DSCOVR/EPIC SO_2_ imagery is available from the Earthdata archive (https://www.earthdata.nasa.gov/). The GFS meteorological data is available from NOAA (ftp://arlftp.arlhq.noaa.gov/pub/archives/). All radiosonde data was taken from the University of Wyoming Department of Atmospheric Science website (https://weather.uwyo.edu/upperair/sounding.shtml). All PlumeTraj results presented in this paper are available online (10.48420/28070294).
