# Supplementary material for: Insights into eruption dynamics from TROPOMI/PlumeTraj-derived SO2 emissions during the 2022 eruption of Mauna Loa, Hawaiʻi
Source: Bull Volcanol. 2025 Aug 14;87(9):69. doi: 10.1007/s00445-025-01839-8 (PMC12354495; doi:10.1007/s00445-025-01839-8)
Supplement: Supplementary file 1 — (PDF 1.25 MB) [file 445_2025_1839_MOESM1_ESM.pdf]

Insights into eruption dynamics from TROPOMI/PlumeTraj-derived SO_2 emissions during the 2022 eruption of Mauna Loa, Hawaiʻi – Supplementary Information

B. Esse[1], M. Burton[1], H. Brenot[2], N. Theys[2]

[1] COMET, Department of Earth and Environmental Sciences, The University of Manchester, Manchester, UK

[2] Royal Belgian Institute for Space Aeronomy, Brussels, Belgium

Corresponding author: B. Esse, benjamin.esse@manchester.ac.uk

This file contains the supplementary figures (S1 – 6) for this article.

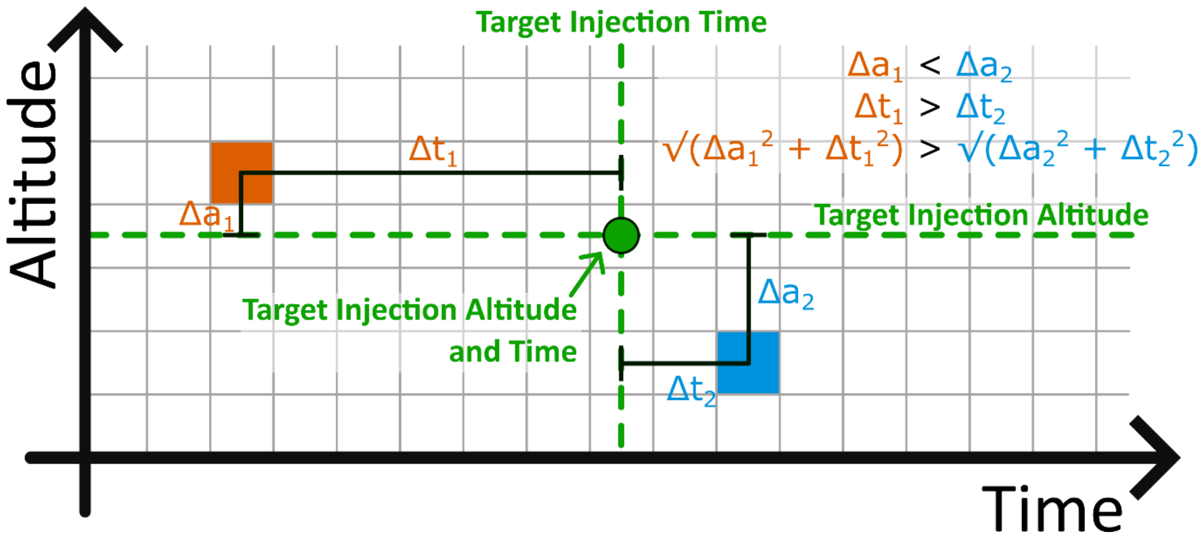

Fig. S1. Example of PlumeTraj solution selection for a pixel based on target injection values (in green) compared to two possible solutions. If using just the target injection altitude, then solution 1 (orange) will be selected, however if using either the target injection time or the target injection altitude and time then solution 2 (blue) will be selected.

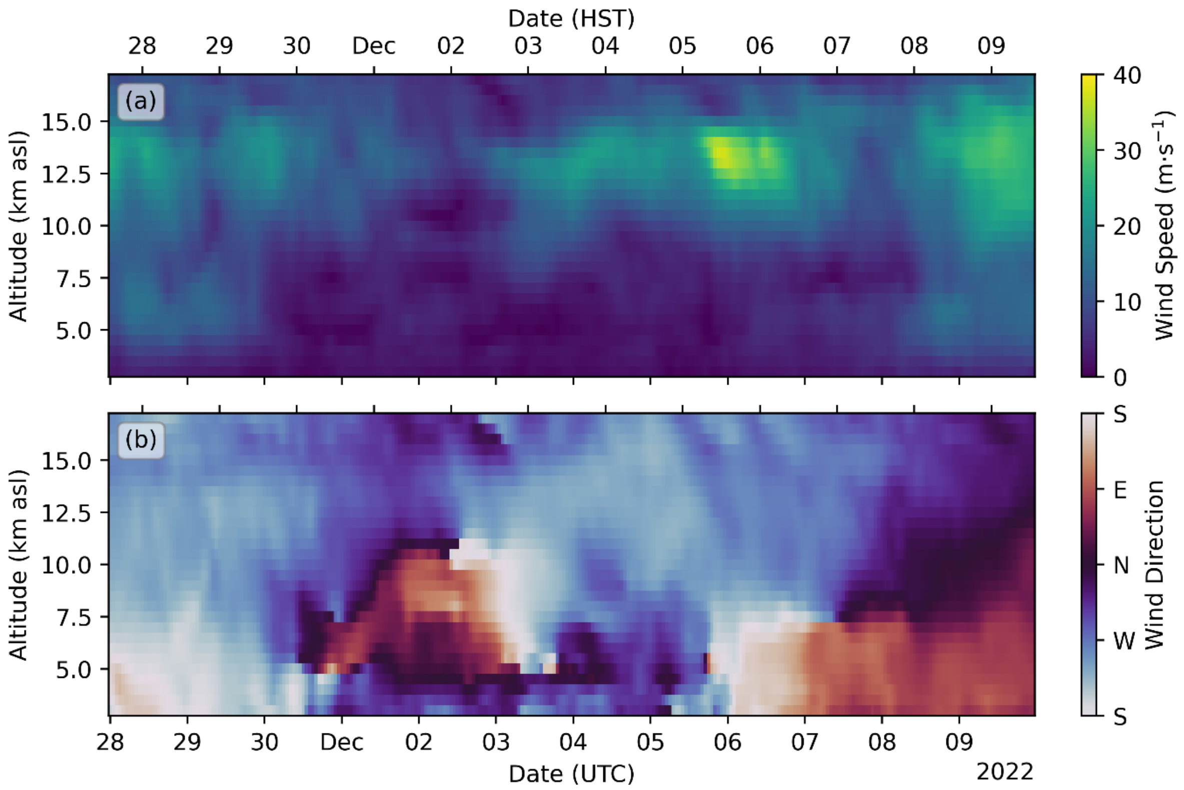

Fig. S2. Reconstructed wind data above the summit vent (19.475° N, 155.608° W) from GFS model data, showing (a) the wind speed and (b) the wind direction.

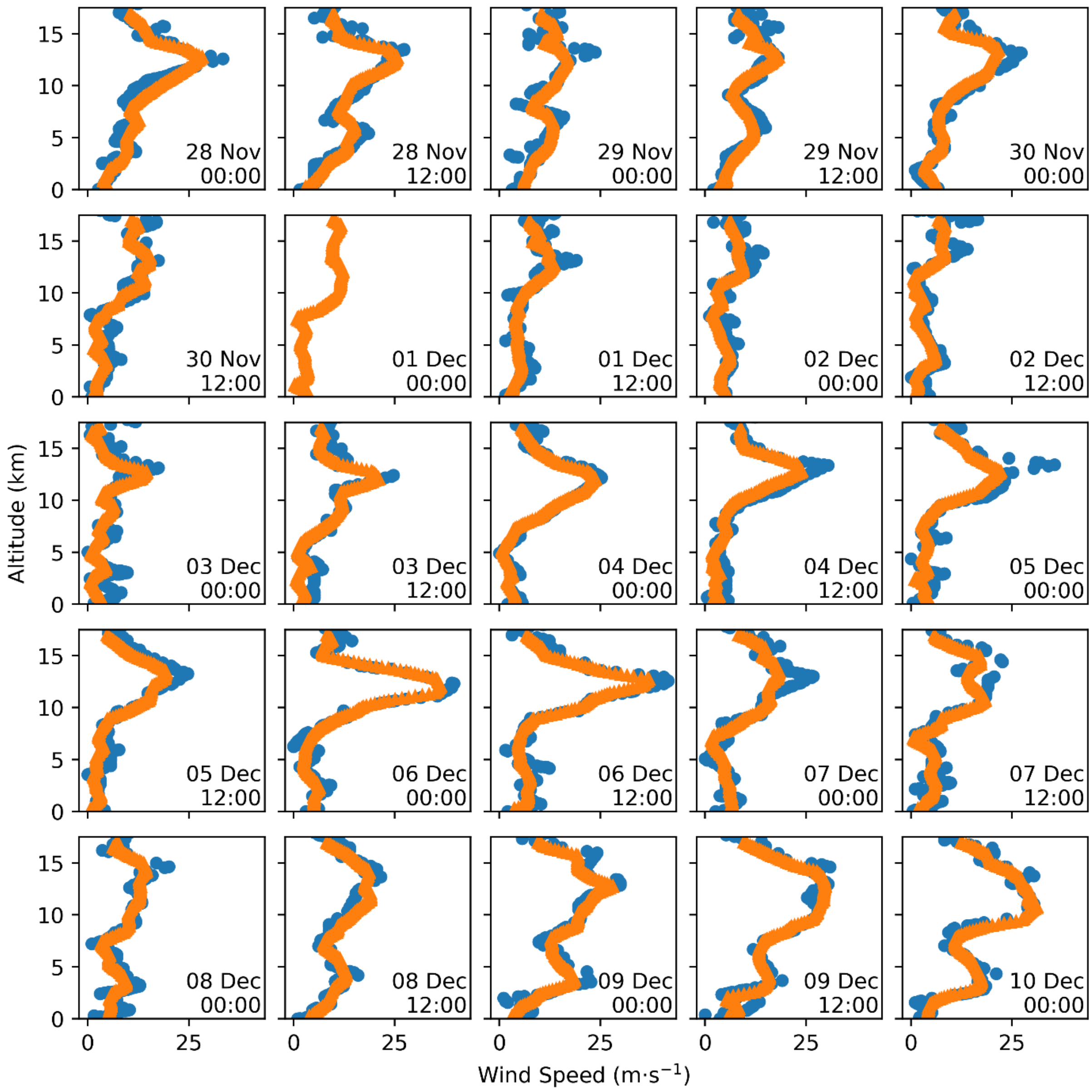

Fig. S3. Comparison of wind speeds between PHTO station radiosonde launches (blue circles) and the GFS model above the launch location (19.72 °N, 155.05 °W , orange triangles) across the eruption. Note the launch at 2022-12-01 00:00 failed.

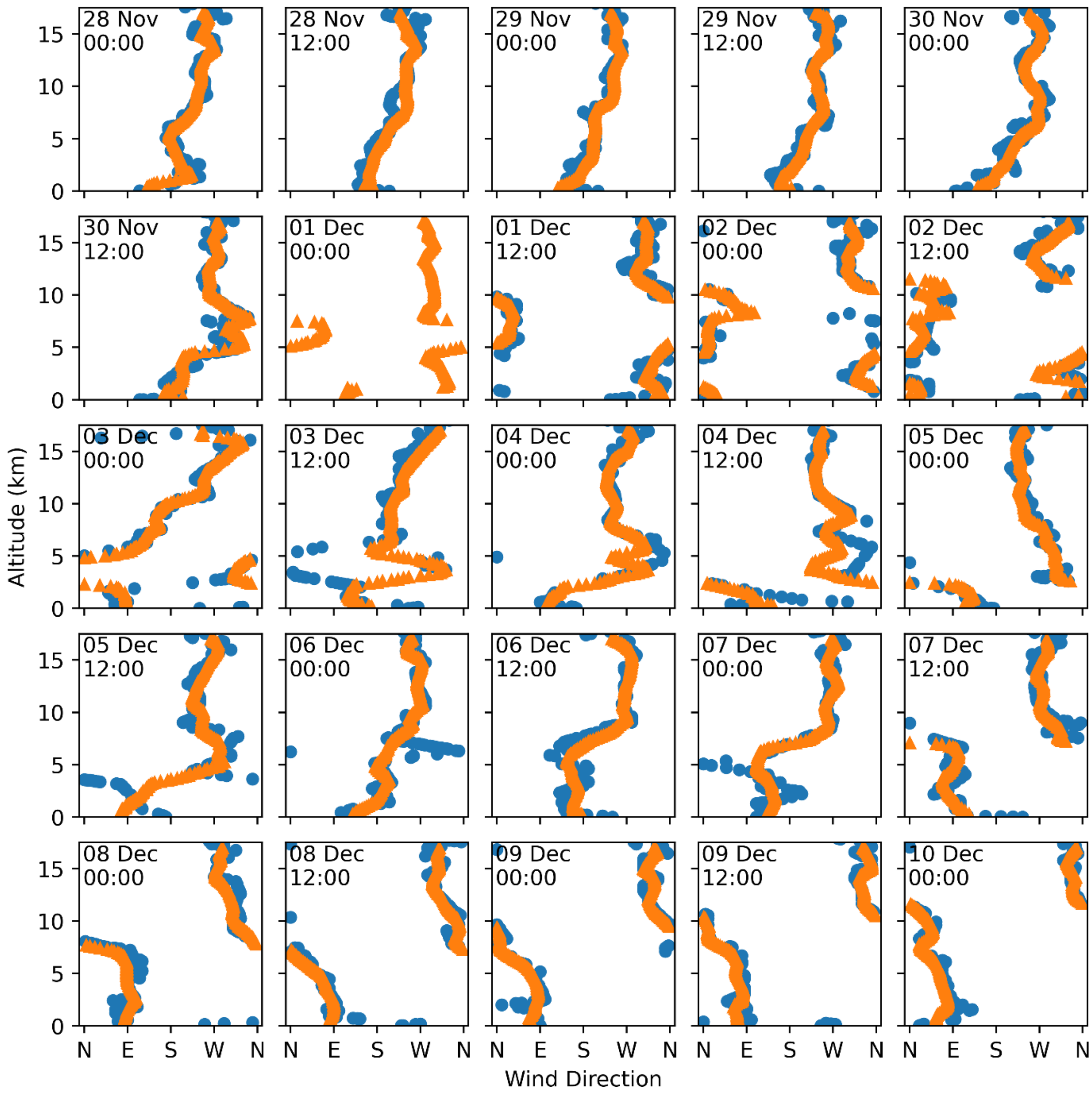

Fig. S4. Comparison of wind directions between PHTO station radiosonde launches (blue circles) and the GFS model above the launch location (19.72 °N, 155.05 °W, orange triangles) across the eruption. Note the launch at 2022-12-01 00:00 failed.

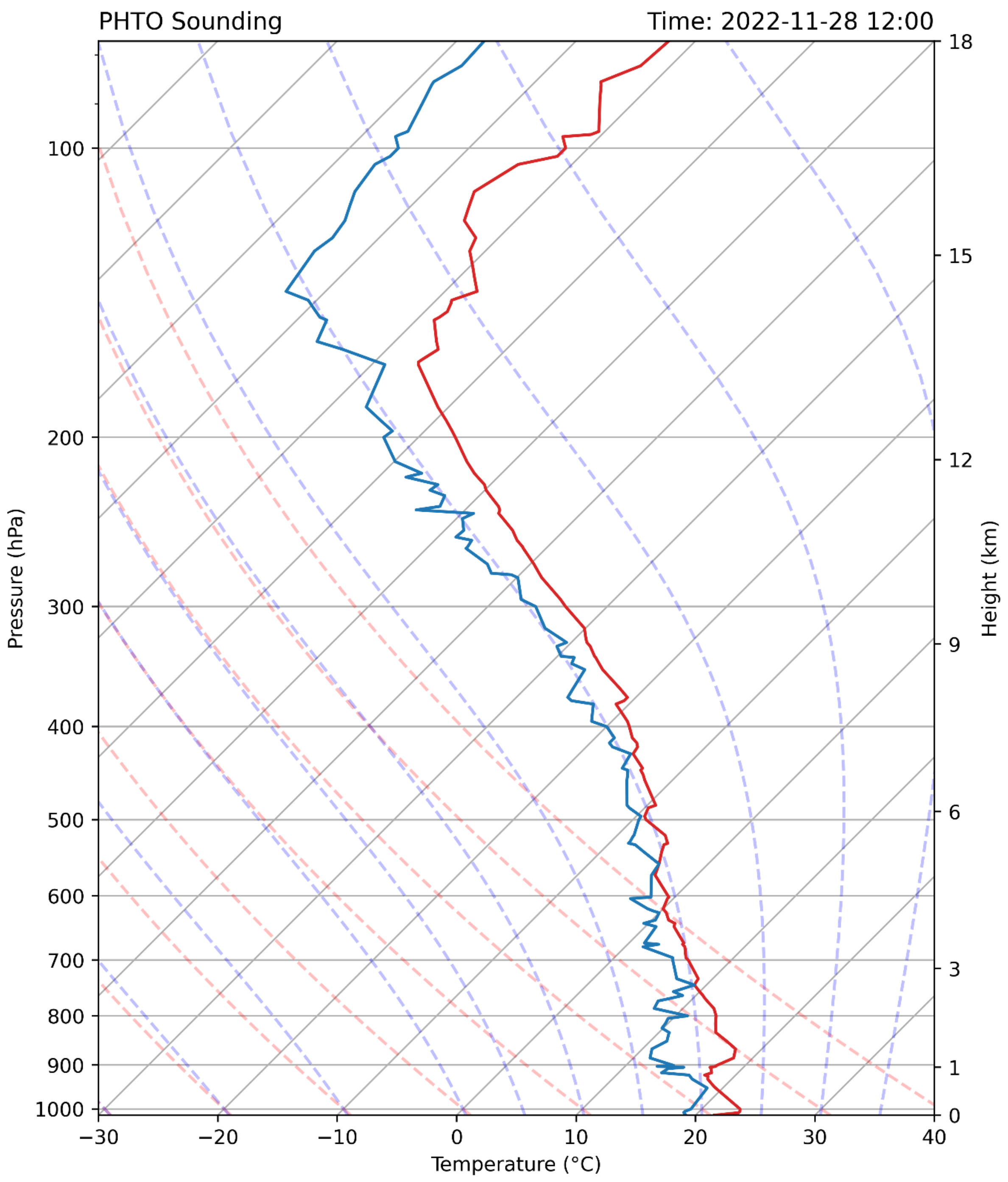

Fig. S5. Skew-T plot from radiosonde sounding at 12:00 on 28th November (UTC) from the PHTO station (19.72 °N, 155.05 °W), showing the temperature (red) and dew-point (blue) as a function of altitude. The altitude of the tropopause is roughly 14 – 16 km. Data from the University of Wyoming Department of Atmospheric Science (https://weather.uwyo.edu/upperair/sounding.html).

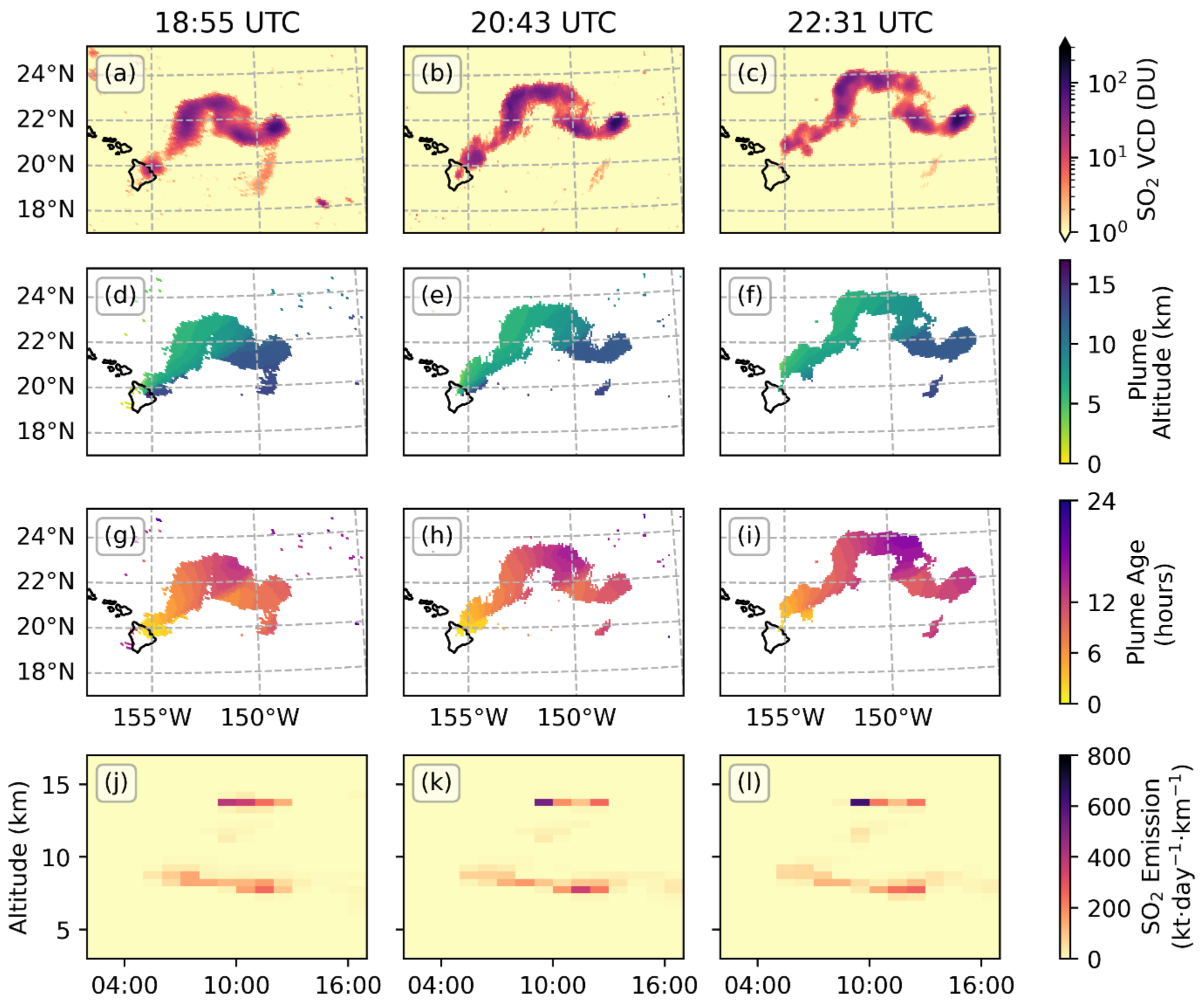

Fig. S6. SO_2 VCDs measured by the EPIC instrument onboard DSCOVR (a-c), PlumeTraj derived plume altitude (d-f) and ages (g-i), and SO_2 emissions (j-l). Measurement time is given at the top of each column. Times in UTC.
